# Fine-mapping and identification of candidate causal genes for tail length in the Merinolandschaf breed

**DOI:** 10.1038/s42003-022-03854-3

**Published:** 2022-09-06

**Authors:** Dominik Karl Lagler, Elisabeth Hannemann, Kim Eck, Jürgen Klawatsch, Doris Seichter, Ingolf Russ, Christian Mendel, Gesine Lühken, Stefan Krebs, Helmut Blum, Maulik Upadhyay, Ivica Medugorac

**Affiliations:** 1 Population Genomics Group, Department of Veterinary Sciences, LMU Munich, Lena-Christ-Str. 48, 82152 Martinsried, Germany; 2 Tierzuchtforschung e.V. München, Senator-Gerauer-Str. 23, 85586 Poing, Germany; 3 Institute for Animal Breeding, Bavarian State Research Center for Agriculture, Prof.-Dürrwaechter-Platz 1, 85586 Poing, Germany; 4 Institute of Animal Breeding and Genetics, JLU Gießen, Ludwigstr. 21, 35390 Gießen, Germany; 5 Laboratory for Functional Genome Analysis, Gene Center, Ludwig-Maximilians-University Munich, 80539 Munich, Germany

**Keywords:** Animal breeding, Structural variation

## Abstract

Docking the tails of lambs in long-tailed sheep breeds is a common practice worldwide. But this practice is associated with pain. Breeding for a shorter tail could offer an alternative. Therefore, this study aimed to analyze the natural tail length variation in the Merinolandschaf and to identify causal alleles for the short tail phenotype segregating within long-tailed breeds. We used SNP-based association analysis and haplotype-based mapping in 362 genotyped (Illumina OvineSNP50) and phenotyped Merinolandschaf lambs. Genome-wide significant regions were capture sequenced in 48 lambs and comparatively analyzed in various long and short-tailed sheep breeds and wild sheep subspecies. Here we show a SNP located in the first exon of *HOXB13* and a SINE element located in the promotor of *HOXB13* as promising candidates. These results enable more precise breeding towards shorter tails, improve animal welfare by amplification of ancestral alleles and contribute to a better understanding of differential embryonic development.

## Abstract (web summary)

Using SNP-association analysis and genetic mapping, a SNP and an insertion in and close to *HOXB13* associated with short tail length is identified in Merino sheep, which may be a target for safely selecting shorter tails and improving sheep welfare.

## Introduction

Sedentary human communities began sheep management as early as 10,000–11,000 BP in an area stretching from central Anatolia to northwestern Iran^1^. It is proposed that the Asiatic mouflon (*Ovis orientalis*), which was common in the same area, was the wild ancestor. The Asiatic mouflon, like other wild sheep subspecies, is a short-tailed hair sheep. Accordingly, the first domesticated sheep were also short-tailed hair sheep, kept mainly for their meat^2^. The systematic production and processing of wool did not occur until several millennia later, leading to the “Secondary Product Revolution”^3^ and the worldwide replacement of hair sheep by wool sheep. Long-term selection for fine wool fibers culminated in the economically most important and widespread group of sheep breeds, the Merinos. All Merinos are characterized by long tails and the common occurrence of fine wool and a long tail led to the frequent opinion that these phenotypes are also genetically coupled or the result of the same artificial selection^4^. This assumption could not be proven directly, however, in today’s sheep husbandry systems the long woolly tail comes with several problems, e.g., the accumulation of dags in the tail area, which predisposes for flystrike^5^. Therefore, most lambs of long-tailed breeds worldwide are docked shortly after birth^4^. With the increasing importance of animal welfare in our society and subsequent restrictions and prohibitions of practices that cause pain and suffering to animals, tail docking has come under scrutiny. In Scandinavia, tail docking without a veterinary indication has already been made illegal^6^ and in the Netherlands, exclusions from the docking ban have only been granted for three long-tailed English breeds under the condition of an effective breeding program for shorter tails^7^. In Austria, tail docking in lambs is allowed until the age of 7 days, provided the operation is done by a veterinarian or another qualified person and an analgesic for intra- and post-operative pain-relief is given^8^. The German Animal Welfare Act currently still allows tail docking without anesthesia for lambs under eight days of age^9^, but future amendments will probably seek to eliminate exceptions to the amputation ban^10^.

These developments clearly show that a long-term non-invasive alternative for the painful practice of tail docking is urgently needed. Here, a genetic solution offers itself. A high ethical acceptance of genetic breeding for a shorter tail could be expected as all wild sheep subspecies and thus also the ancestor of today’s domestic sheep have naturally short tails^11,12^. Therefore, this breeding could be seen as a “back to the roots” program.

The genetic basis for shorter tail breeding efforts is provided by the medium to high heritability of tail length (TL) in different sheep breeds, e.g., 0.58 in Merinos^13^ or 0.77 in Finnsheep^14^. James et al.^15^ suggest that the inheritance of TL in Australian Merino depends on a small number of interacting genes of large effects, in which short tail genes show a possible dominance. The presence of various short-tailed Nordic breeds offers a possibility of genetic reduction of TL by introgression of the desired genetic variants from short-tailed breeds. Scobie and O’Connell^16^ crossed short-tailed Finnsheep with long-tailed Cheviot sheep and observed that an increased proportion of Finnsheep genes led to a proportional reduction in TL. However, this option is unpopular with breeders, as crossing is associated with the loss of breed-specific traits and a possible decline in previously achieved breeding progress in important production traits^17^. In extreme cases, it could also introduce undesirable traits/conditions in the population. For example, Jordan^18^ described fully or partially hindquarter paralysis in the No-tail lambs from the cross of Siberian fat-rumped sheep with other sheep breeds such as Hampshire sheep and Rambouillet sheep. This condition is attributed to the appearance of a lethal gene, known as “sidewheeler”. The majority of the lambs with this condition starved to death during early years of their life. Post-mortem examination of these lambs revealed the abnormal termination of the spinal cord.

As an alternative, breeders in Australia and New Zealand attempted genetic shortening of the tail by phenotypic selection within individual breeds. However, Carter^19^ reported for Romney sheep that breeding for the short-tail phenotype possibly reduced the viability of embryos that were homozygous carriers of some putative short-tail alleles. In Merinos, James et al.^15^ observed increased incidences of rear-end defects. Zhi et al.^20^ discovered a c.G334T mutation in the *T* gene in the native Chinese Hulunbuir breed and showed that the T allele leads to the extreme short-tailed phenotype, i.e., tailless animals with exposed anus. To prove the causality of this mutation, they genotyped 120 short-tailed Hulunbuir sheep. The observed frequencies of the genotypes (17 G/G, 103 G/T, and no T/T) are consistent with the embryonic lethality due to the T/T genotypes in *T* gene^20^. On the other hand, Han et al.^21^ claim that the nonsynonymous c.334 G > T mutation is predominantly or exclusively homozygous in Chinese fat-rumped and Hulunbuir sheep. Moreover, this genotype also segregates in other breeds and is always associated with the synonymous c.333 G > C mutation. Despite these two contradictory statements by Zhi et al.^20^ and Han et al.^21^, both studies concluded that an association between the (extremely) short tail phenotype and the c.334 G > T mutation is likely. A comparable association between short tails and embryonic lethality or malformations has been demonstrated in various breeds of dogs and cats too^22–24^. These undesirable negative side effects discouraged and slowed down active breeding programs against overlong tails in the economically most important wool sheep breeds. Moreover, there have been no successful genetic mapping studies in long-tailed Merino sheep breeds and the possible relationship between the short tail phenotype and embryonic viability or hind end malformations has, to the best of our knowledge, never been investigated on a genetic basis in Merinos.

The aim of the present study was therefore:

1. To investigate the phenotypic and additive-genetic variance in TL in the Merinolandschaf, which belongs to the economically most important long-tailed Merino breed group worldwide;
2. To map the position of the major quantitative trait locus (QTL) affecting TL;
3. To detect and confirm causal candidate genes by sequencing and genotyping;
4. To determine the distribution of ancestral and derived alleles in a wide range of domestic sheep breeds with different TLs as well as in different wild sheep subspecies;
5. To contribute to the understanding of the relationship between genotype and phenotype during embryonic patterning and early development;
6. To put causal alleles in the evolutionary context of sheep species.

Together, these objectives will enable more efficient breeding towards the ancestral phenotype and thus improve animal welfare in sheep production without negative side effects.

## Results

### Initial mixed linear model association analysis

**Fig. 1:**
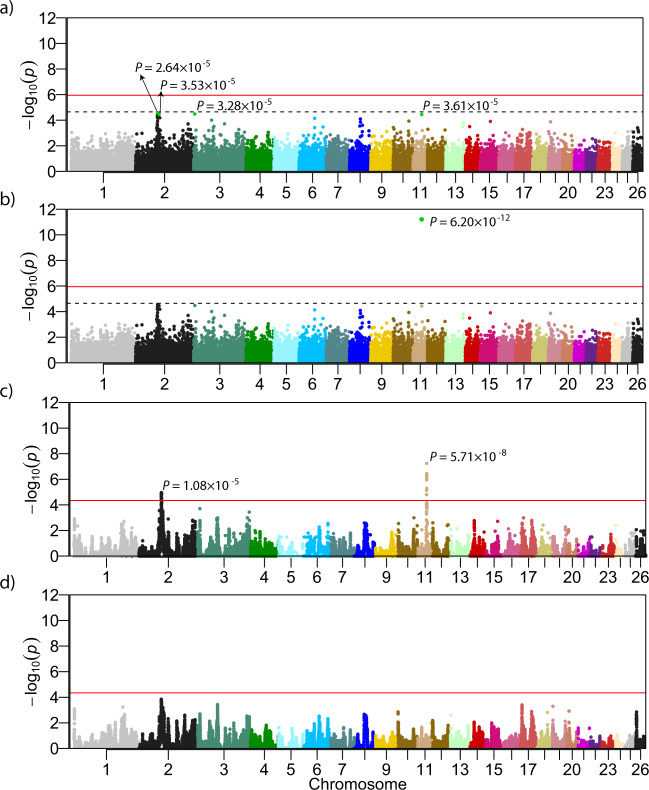
Results of performed mixed linear analyses presented as Manhattan plots. **a** MLMA Model 1 with 45,114 markers, no marker were above the suggestive line, the four markers with the lowest *p* values are shown; **b** MLMA Model 2 with 45,115 markers, the additionally added candidate locus on OAR11 shows genome-wide significance; **c** cLDLA Model 3 with 45,114 markers resulted in two genome-wide significant peaks on OAR2 and OAR11; **d** cLDLA Model 5 with 45,115 markers and candidate locus added as fixed effect, the peak on OAR2 decreases below the genome-wide significance and the peak on OAR11 erases completely.

*GCTA-GREML* analysis revealed SNP-based heritability of 0.992 (standard error of 0.12), meaning that a very high proportion of the TL variance in Merinolandschaf breed is explained by genome-wide SNP markers. Despite this very high heritability, i.e., close to 1, the initial association analysis (MLMA Model 1, Table 1) revealed no genome-wide significant association between any SNPs and TL. Even the four most significant SNPs (Fig. 1a) remain below the suggestive significance threshold of *P* = 2.22 × 10^−5^.

**Table 1:** Mixed linear models used for association and combined linkage disequilibrium and linkage analysis.

Analysis	Model name	Effects		Comment
		Fix	Random	
MLMA	Model 1	µ, sex, age, BW, WH, a	u, e	
	Model 2	µ, sex, age, BW, WH, a	u, e	add one candidate locus as marker
cLDLA	Model 3	µ, sex, age, BW, WH	u, q, e	
	Model 4	µ, sex, age, BW, WH	u, q, e	add one candidate locus as marker
	Model 5	µ, sex, age, BW, WH, cSNP, a	u, q, e	add one candidate locus as fixed effect

The fixed and random effects are the overall mean (µ), sex, age, body weight (BW), withers height (WH), the vector of the additive effect of the candidate marker to be tested for association (a), the vector of random polygenic effects (u), the vector of random additive-genetic QTL effects (q) and the vector of random residual effects (e).

### Initial combined linkage disequilibrium and linkage analysis

The haplotype-based cLDLA mapping (cLDLA Model 3) resulted in two genome-wide significant QTLs associated with TL in Merinolandschaf (Fig. 1c). The most prominent and narrow peak is on OAR11 at position 37,111,462 bp with *LRTmax* = 29.460 corresponding to *P* = 5.71 × 10^−8^ (Bonferoni corrected: *P* = 6.43 × 10^−5^) as shown in Supplementary Fig. S1a. The second genome-wide significant QTL affecting TL was mapped to the OAR2 at position 94,538,115 bp with *LRTmax* = 19.356 corresponding to *P* = 1.08 × 10^−5^ (Bonferoni corrected: *P* = 1.22 × 10^−2^).

Applying the 2-LOD criterion, the corresponding confidence interval (CI) was set for the *LRTmax* on OAR11 between positions 37,000,925 bp and 37,521,490 bp and for the maximum value on OAR2 between positions 93,441,900 bp and 96,402,884 bp. These intervals were then considered in the UCSC Genome Browser Oar_v4.0/oviAri4 Assembly, which resulted in the list of genes summarized in Table 2 and Table 3. The list of positional candidates also includes obvious functional candidates from the sheep homeobox B gene cluster (Chr11:37,290,203–37,460,240) with *HOXB13* (37,290,203–37,292,513) as the closest and most prominent candidate, lying only 179 Kb proximal to the *LRTmax*.

**Table 2:** Genes on OAR2 between positions 93,441,900 bp and 96,402,884 bp as well as the genome-wide significant peak of cLDLA Model 3.

Gene	Name	Start (bp)	End (bp)
*CAAP1*	Caspase activity and apoptosis inhibitor 1	94,480,790	94,556,081
QTL-Peak	Peak of cLDLA Model 3	94,538,115	
*PLAA*	Phospholipase A2 activating protein	94,567,698	94,605,168
*IFT74*	Intraflagellar transport 74	94,607,523	94,708,596
*LRRC19*	Leucine-rich repeat containing 19	94,629,884	94,643,326
*TEK*	TEK receptor tyrosine kinase	94,739,266	94,838,005
*EQTN*	Equatorin	94,900,676	94,917,170
*MOB3B*	MOB kinase activator 3B	94,934,882	95,077,788
*IFNK*	Interferon kappa	95,145,920	95,147,256
*C9ORF72*	C9orf72-SMCR8 complex subunit	95,183,641	95,202,857
*LINGO2*	Leucine rich repeat and Ig domain containing 2	95,627,231	95,629,051

**Table 3:** Genes on OAR11 between positions 37,000,925 bp and 37,521,490 bp as well as the genome-wide significant peaks of cLDLA model 3 and 4.

Gene	Name	Start (bp)	End (bp)
*IGF2BP1*	Insulin-like growth factor 2 mRNA binding protein 1	37,009,695	37,032,963
*GIP*	Gastric inhibitory polypeptide	37,074,497	37,079,791
*SNF8*	SNF8, ESCRT-II complex subunit	37,093,635	37,101,565
*UBE2Z*	Ubiquitin conjugating enzyme E2 Z	37,104,244	37,117,532
QTL-Peak	Peak of cLDLA Model 3	37,111,462	
*ATP5G1*	ATP synthase	37,125,809	37,128,277
*CALCOCO2*	Calcium binding and coiled-coil domain 2	37,179,738	37,202,548
*TTLL6*	Tubulin tyrosine ligase like 6	37,221,445	37,263,708
*HOXB13*	Homeobox B13	37,290,203	37,292,513
QTL-Peak	Peak of cLDLA Model 4	37,311,842	
*HOXB9*	Homeobox B9	37,365,122	37,369,564
*HOXB8*	Homeobox B8	37,376,709	37,378,201
*HOXB7*	Homeobox B7	37,381,261	37,384,010
*HOXB6*	Homeobox B6	37,391,399	37,395,989
*HOXB5*	Homeobox B5	37,397,814	37,404,968
*HOXB3*	Homeobox B3	37,437,808	37,439,623
*HOXB2*	Homeobox B2	37,445,188	37,448,075
*HOXB1*	Homeobox B1	37,457,621	37,460,240

Additional chromosome-wide significant peaks were observed on OAR2, OAR3, OAR10, OAR14, and OAR17. However, these peaks show *LRT* values far below the genome-wide significance and thus, were not investigated further.

### Estimation of QTL effects and selection of animals for capture sequencing

In the previous step of cLDLA, we used *ASReml*^25^ to estimate variance components, fixed and random effects affecting TL in Merinolandschaf breed. Here, we analyzed in more detail the estimated effects at loci with the most significant association, i.e., at loci showing *LRTmax* values. We sorted all 362 lambs according to the random additive-genetic QTL effects (vector **q**) estimated at *LRTmax*. Together with the QTL effects, we simultaneously considered all input (**y**, sex, age, body weight (BW), withers height (WH), maternal and paternal haplotypes at 40-SNP window with *LRTmax* in interval between SNP 20 and 21) and output data (**u**, **ß**, and **e**) that contributed to *LRTmax*. Visual inspection of this table and regression analysis allowed us to select 48 lambs for targeted capture sequencing. Supplementary Figs. S2, S3 show the distribution of the sequenced lambs regarding TL and diplotype effects on OAR2 and OAR11.

A regression analysis performed with the function *lm* in *R*^26^ estimated the adjusted coefficient of determination of *R*^*2*^ = 0.58 for the *LRTmax* on OAR11 and only *R*^*2*^ = 0.15 for the *LRTmax* on OAR2 when using TL as the dependent and diplotype effect as the independent variable. Adding, age, sex, BW, and WH as additional independent variables yield *R*^*2*^ = 0.78 for QTL on OAR11 and *R*^*2*^ = 0.45 for QTL on OAR2 (see Supplementary Tables S1, S2). According to the shape of the LRT curve, the significance of the mapping and the coefficient of determination, the haplotypes associated with the putative causative alleles are more distinct in QTL on OAR11 than on OAR2. However, the selection of 48 lambs for capture sequencing represents a trade-off between the two QTLs, with the choice for OAR11 being more decisive. The selected lambs could be divided into two groups: 23 long-tailed with positive QTL effect and 25 short-tailed lambs with negative QTL effect on OAR11. Sorting the same lambs by QTL effects on OAR2 changes the order within the group and results in 3 individuals from the long-tailed group and 4 individuals from the short-tailed group moving to the other group.

### Capture sequencing of 48 lambs and detection of candidate mutations

Capture Sequencing was carried out at a mean depth between 0.41 and 2.45 on the target region of OAR2 and between 1.01 and 2.39 on the target region of OAR11. This coverage is much lower than intended and most possibly caused by competition with whole-genome sequencing (WGS) performed on the same sequencing lane. However, applying default settings of GATK *HaplotypeCaller*^27^, we detected 40,433 short variants on the investigated region on OAR11. Next, we sought to identify variants showing remarkable differences in frequency between short-tailed and long-tailed groups by applying 5 steps described in supplementary methods. This allowed us to reduce the number of potential candidates from 40,433 to 19 (Supplementary Table S3). Of all these 19 most plausible genomic variations, only one SNP nearly satisfied the frequency-based criterion (see Supplementary Methods). Interestingly, this SNP (C→G) was located at position 37,290,361. The visual examination using *Jbrowse*^28^ confirmed the base substitution (*rs413316737*) as a nonsynonymous point mutation within the first exon of *HOXB13* (relative position 23). The point mutation results in a p.(Thr8Ser) substitution. All sequenced Merino lambs from the long-tailed group are homozygous for this missense variant (G/G). In the short-tailed group, 4 lambs are homozygous G/G, 6 are heterozygous C/G and 15 are homozygous C/C on that position. The Ensembl Variant Effect Predictor^29^ predicted a SIFT score of 0.54 and classified the mutation as so-called “*tolerated*” missense variant.

**Fig. 2:**
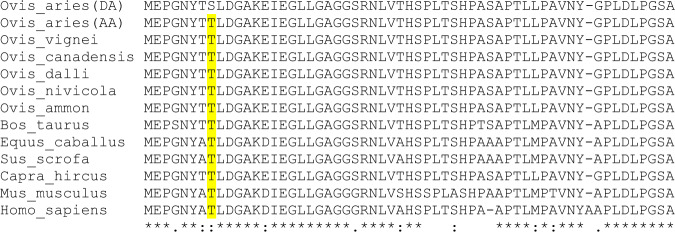
Alignment of the initial amino acid sequence of HOXB13 in different mammals. Positions of interest are highlighted. The asterisks present unique, the colons high similar and the single point moderate similar amino acids in every species on the respective position. To highlight the area of interest we used Clustal Omega^90,91^ DA derived allele, AA ancestral allele.

In the next step, protein *BLAST*^30^ was used to align the amino acid sequence of the mutant HOXB13 protein against the amino-acid sequence of wild-type HOXB13 protein of different mammals including all the extant wild sheep species shown in Supplementary Table S4. This cross-species alignment (Fig. 2) revealed that the amino acid at which the discovered variant occurred is conserved. In addition, 5 downstream amino acids are also conserved among the aligned species. By comparing the allele frequency in long-tailed and short-tailed sheep in multiple breeds, we observed that the above-mentioned derived allele G occurs more frequently in long-tailed sheep breeds (Supplementary Table S5). Further, at this position, we observed only the ancestral allele in Urial, Argali, Snow sheep, Dall sheep, Canadensis and two ancient (~8000 years) sheep genomes^31^. On the other side out of 16 investigated Asiatic mouflon 3 were heterozygous C/G and 2 homozygous G/G for the point mutation. However, it is worth mentioning here that one Asiatic mouflon (G/G) and the two ancient WGS have low coverage at this locus.

**Fig. 3:**
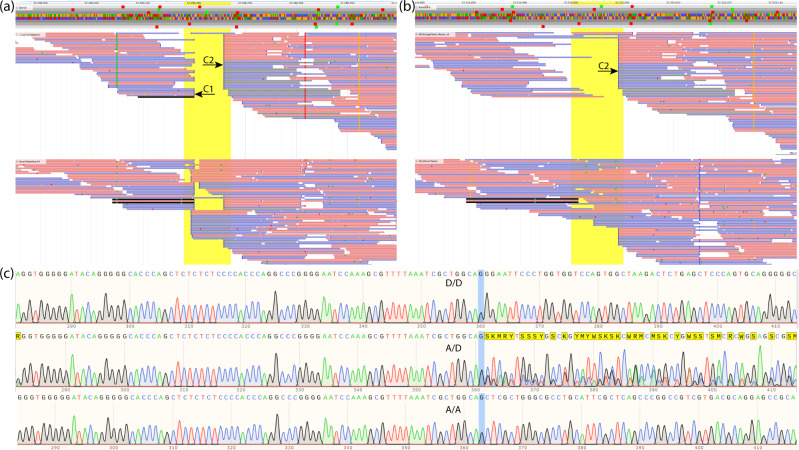
Screenshots showing insertions, position of the candidate SNP at position 37,290,631 on OAR11, and extracts from ab1 trace files from Sanger sequencing. **a** Reads of the pooled sequenced long-tailed (above) and short-tailed (below) Merinolandschaf lambs mapped on assembly Oar_4.0, note the two clusters, cluster one (C1) is likely due to an assembly problem, cluster two (C2) represents an insertion shown in 34 of the 35 reads in the long-tailed group and in 13 of the 54 reads in the short-tailed group; **b** shows the same groups mapped against the newest assembly ARS-UI_Ramb_v2.0, note the disappearance of C1; **c** Sanger sequences for one homozygous derived (D/D), one heterozygous (A/D) and one homozygous ancestral (A/A) lamb around 40 bp before and after the breakpoint of the real insertion.

Further, we identified SVs in the targeted region of OAR11 by using three different approaches: *Smoove*^32^, *Delly*^33^, and visual examination with *Jbrowse*. Using the strict threshold criteria, i.e., SV not present in a pooled sample of short tail sheep but present in a pooled sample of long tail sheep, we identified 27 and 32 SVs from *Smoove* and *Delly* approaches, respectively. However, it is noteworthy that the captured sequencing had highly non-uniform and relatively low coverage, therefore, these approaches might have missed many true positives and included high number of false positives. In fact, the visual examination of these regions also indicated so (Fig. 3).

Interestingly, window-by-window visual examination of the targeted region in *Jbrowse* revealed two distinct clusters of soft-clipped reads (Fig. 3a) just about ~130 bp upstream of the candidate SNP. Both clusters were arranged next to each other. One cluster was present in all sequenced animals, indicating either assembly error or assembly-specific variant as the likely cause. The other cluster of soft-clipped reads had distinct frequency distribution between the groups of short-tailed and long-tailed reads. At position 37,290,229 on OAR11, the long-tailed group showed 34 of the 35 reads mapped as soft-clipped, while the short-tailed group showed 13 of the 54 reads as soft-clipped. We also observed a remarkable difference in the frequency of the clipped reads around this position between the WGS data of short and long-tailed sheep that were downloaded from NCBI SRA (Supplementary Table S4). Therefore, in the next step, we aligned the pooled reads of the short-tailed and the long-tailed group and the WGS data of NCBI SRA against the latest sheep assembly (ARS-UI_Ramb_v2.0)^34^ (https://www.ncbi.nlm.nih.gov/assembly/GCF_016772045.1). Visual examination of the region upstream of the first exon of *HOXB13* gene on OAR11 revealed only one cluster of soft-clipped reads at positions 37,524,996 (Fig. 3b) indicating that another cluster which was identified in the mapping against Oar 4.0 assembly was due to missing sequences of about 40 bp in Oar 4.0 assembly. Interestingly, we observed that a very high number of soft-clipped reads in this cluster had supplementary alignment on OAR5. Further, on OAR11 at the breakpoint, we also observed discordant alignment (overlapped) between forward and reverse reads. Based on the presence of the soft-clipped reads and discordant alignment, we suspected the presence of an insertion or translocation. While we were investigating this region on ARS-UI_Ramb_v2.0 assembly, we came across a pre-print by Li et al.^35^ who reported an insertion in the same region.

To investigate the soft-clipped regions further, we carried out Sanger sequencing of five samples, based on the previously described candidate SNPs. The analysis of the Sanger sequencing data (Fig. 3c) and subsequent alignment against OAR11 in ARS-UI_Ramb_v2.0 using *BLAST*^30^ identified the SV as an insertion of 167 bp. We further observed that this sequence is flanked by 14 bp of direct repeats (CTGCCAGCGATTTA) on both sides. Therefore, we hypothesized that this insertion could be a part of short interspersed nuclear elements (SINE) repeat family. Next, we searched for this sequence in DFAM repeat database and identified it as belonging to OviAri-1.113 SINE family.

### Genotyping of the most plausible candidates in 362 lambs and remapping of tail length

To confirm the association between the detected variants and TL, we performed genotyping of these two candidates in the entire mapping population and used genotypes in the GWAS and cLDLA Model 2, 4, and 5 (Table 1). The PCR-genotyping of the candidate SNP resulted in 220 G/G, 118 C/G, and 24 C/C lambs. The PCR-genotyping of the 132 bp upstream candidate insertion of 167 bp showed an identical distribution of genotypes over the entire mapping population, i.e., the insertion occurred in all haplotypes harboring the base G and never in haplotypes harboring C on the position 37,290,361. Due to the complete linkage between the insertion and the missense SNP, both candidates are considered synchronously and equally in Model 2 (MLMA) and Models 4 and 5 (cLDLA). The distribution of alleles in wild sheep subspecies and domestic sheep allows us to infer ancestral and derived alleles at both candidate loci. The absence of the insertion and presence of base C at c.C23G SNP in *HOXB13* are ancestral alleles, whereas the 167 bp insertion and base G indicate derived alleles.

All lambs homozygous for ancestral alleles could be classified as short-tailed with mean tail-length of 24.1 cm (±1.34) and mean QTL effect of −2.92 cm (±0.71). On the other side lambs homozygous for derived alleles classified as long-tailed as well as short-tailed. Consequently, lambs with derived homozygous genotype show higher average TL of 31.5 cm and 4.15 times higher standard deviation of TL (±5.56). Lambs with heterozygous genotype show average TL of 25.7 and 2.73 times higher standard deviation (±3.66). Table 4 summarizes phenotype, QTL, and polygenic effects of candidate insertion and SNP that are in population-wide linkage disequilibrium.

**Table 4:** The number of individuals, mean and SD of tail length, QTL effects, and polygenic effects of the different genotype groups.

Genotype	Count			Tail length		QTL effect		Polygenic effect	
	All	Short	Long	Mean	SD	Mean	SD	Mean	SD
D/D	220	70	150	31.5	5.56	0.68	1.27	1.12	3.56
A/D	118	94	24	25.7	3.66	−1.3	0.89	−1.61	2.96
A/A	24	24	0	24.1	1.34	−2.92	0.71	−2.19	1.52

The groups are homozygous ancestral (A/A), homozygous derived (D/D), and heterozygous (A/D).

### MLMA and cLDLA including candidate variants as markers

To investigate the impact of derived alleles on the results of the SNP-based association analysis and the haplotype-based mapping, we considered the candidate locus as an additional marker located on Chr11:37,290,361 (see Model 2 and 4 Table 1). Thereby, only the number of considered markers increases by one compared to the original analysis, while the other input data, as well as the parameters and the model, do not change. This minimal change in the input data led to enormous changes in the results of the association analysis and limited changes in the results of the haplotype-based mapping. MLMA Model 2 confirmed the candidate causal locus as a uniquely genome-wide significant (*P* = 6.2 × 10^−12^) locus, while cLDLA revealed a slightly altered significance (*LRTmax* = 29.112) at the slightly altered position 37,311,842 bp. However, in the initial mapping *LRTmax* was 179 Kb away from *HOXB13* and the Model 4 of cLDLA placed *LRTmax* between *HOXB13* and *HOXB9* (Table 3). Therefore, the distance between *LRTmax* and the candidate gene *HOXB13* decreased from 179 Kb to 19 Kb and thus *HOXB13* became the closest gene to *LRTmax*.

### Variance components, MLMA, and cLDLA including candidates as fixed effect

The above results point to *rs413316737* and/or the insertion as plausible candidates for causal variations. The mixed linear model (MLMA or cLDLA) allows us to model important causal candidates and thus improve the mapping of residual variance (if present) in the mapping population. To investigate the presence of additional loci affecting TL in long-tailed Merino sheep, we modeled the genotypes at the candidate insertion and SNP as fixed effects, i.e., lambs with homozygous ancestral genotype were classified as class 1, heterozygous as class 2, and homozygous derived as class 3, while the other input data, parameters, and model did not change. We first estimated the SNP-based heritability of TL after correcting for the most significant causal candidates. The heritability decreased from *h*^*2*^ = 0.992 to 0.898. However, as shown in Fig. 1d, the cLDLA is unable to highlight additional candidates, although a relatively high proportion of additive-genetic variance is still present in the mapping population studied here. As expected from the true candidate, the mapping signal on OAR11 disappeared completely (Supplementary Fig. S1b and Fig. 1d). Moreover, the LRT value at the peak on OAR2 decreased from 19.356 to 14.476 and marginally changed its position from 94,538,115 bp to 94,345,619 bp. With this change, a possible QTL on OAR2 loses its genome-wide significance. The mapping results for the region between 35 Mb and 40 Mb on OAR 11 for the models 1, 2, 3, and 5 are shown in Supplementary Fig. S4.

## Discussion

The present study aimed to identify the genes or variants responsible for the natural variability of TL in the Merinolandschaf breed. The results presented here suggest that the inheritance of TL depends largely on additive-genetic variance almost without environment effects (*h*^*2*^ = 0.992). Despite the relatively small number (362) of phenotyped and genotyped animals, heritability was estimated with a relatively low standard error (SE = 0.12). According to Visscher et al.^36^, the SE depends only on the sample size and became below 0.1 by using more than ~3000 independent individuals. Phenotypic correlation in related individuals is often due to a shared environment rather than SNP-associated genetic effects, leading to inflated estimates of heritability^37,38^. Since all the lambs studied here are from only two different farms, the actual heritability is most likely lower than the calculated one, even when relatedness and population structure are taken into account.

The GWAS (MLMA), i.e., the standard method for mapping loci associated with complex traits and diseases, showed no significant and even no suggestive association in the design with 362 animals and 45,114 markers. Typically, GWAS^36^ uses several hundred thousand markers in large mapping designs. Therefore, the solution would be to enlarge the sample size and genotype this enlarged mapping design with high marker density (e.g., OvineHD). However, in studies such as this, where phenotypes are not routinely collected, the number of phenotyped animals may be limited or can only be expanded at great expense. On the other hand, the number of markers can also be a limiting factor in many species, e.g., there is only 50 K chip for domestic goats. The alternative solution could be to apply a mapping method that uses more information from the current design. Due to time and cost constraints, we opted for haplotype-based mapping and obtained a highly significant (*P* = 5.71 × 10^−8^) and fine mapped QTL on OAR11 (CI 37,000,925–37,521,490 Mb) as well as another genome-wide significant result (*P* = 1.08 × 10^−5^) with less sharp mapping (OAR2, CI 93,441,900–96,402,884 Mb). This shows that variance component analysis in the haplotype-based mixed linear model can be more successful than methods such as GWAS when the number of phenotyped individuals and genotyped markers is not large enough for GWAS.

Previous research on TL in domesticated animals, including sheep, mainly pointed to the *T* gene, also known as the brachyury gene, as a candidate causal gene. In Hulunbuir short-tailed sheep, a c.G334T mutation in *T* gene is the main cause of the extreme short-tail phenotype^20^. Mice that are heterozygous for mutations in the *T* gene have a short tail and homozygous embryos die in the middle of the gestation^39–41^. In Manx cats, the short-tailed phenotype is caused by naturally occurring mutations in *T* gene^23^, in particular by three 1-bp deletions. The *T* gene has also been associated with the short-tailed phenotype in various dog breeds^22,42,43^. Additionally, *ANKRD11*, *ACVR2B,* and *SFRP2* were detected as plausible candidate genes that could contribute to the reduction in TL in particular dog breeds^42^ and the somite segmentation-related gene *HES7*^44^ in Asian domestic cats.

In this study, we could not detect any increase in the LRT curve in the *T* gene region (OAR8: 87,717,306–87,727,483bp). Furthermore, neither *ANKRD11* (OAR14: 13,810,611–13,882,911bp), *ACVR2B* (OAR19: 11,794,562–11,802,479bp) or *SFRP2* (OAR17: 3,727,698–3,736,241bp) showed a significant increase in the LRT value. *HES7* is located on OAR11 (27,284,897–27,287,414bp) but is 10 Mb away from the QTL CI and was therefore not considered as a candidate gene for TL in the Merinolandschaf breed. The CI on OAR11 includes the complete ovine *HOXB* cluster and seven additional genes (Table 3). Among these genes, a potential influence on TL could be predicted for *HOXB6*, *HOXB8,* and *HOXB13*. Here, *HOXB13* is the gene closest to the maximum *LRT* value, and a corresponding literature search (see Diaz-Cuadros et al.^45^ for a review) indicates this gene as the most likely gene causing the large effect. The literature search for plausible candidates for genes at OAR2 (Table 2) yielded hardly any useful results. We tried to improve the search with MGI Mammalian Phenotype level 4 ontology, a method for classifying and organizing phenotypic information related to mammalian species. After correction for multiple testing (adjusted *P* < 0.05), 29 ontologies were significantly enriched, but we saw no plausible link to TL.

To select the most suitable lambs for capture sequencing of CIs of QTLs on OAR11 and OAR2, we mutually considered haplotypes, phenotypes, fixed effects, and random effects at *LRTmax* of both regions. Again, the visual inspections, as well as linear regression analyses, confirmed QTL on OAR2 as inconclusive. This is evident from adjusted *R*^*2*^ = 0.58 and only 0.15 for TL fitted to QTL of OAR11 and OAR2, respectively.

Consistent with the clues in favor of OAR11 discussed above, capture sequencing revealed one plausible point mutation and one SINE insertion in the QTL region. All Merinolandschaf lambs and their fathers show a complete linkage disequilibrium between these two variants. This is not surprising because these mutations are only 132 base pairs away from each other and recombination in such a short segment should be extremely rare. We further investigated this linkage in some typical long-tailed and short-tailed breeds (Supplementary Table S4). The linkage was confirmed for long-tailed White Swiss Alpine and Rambouillet breeds but we observed the occurrence of the missense mutation without the insertion in some individuals of short-tailed domestic sheep breeds as well as in five Asiatic Mouflons (Supplementary Table S4). Analyses of the WGS data (Supplementary Table S4) and amino acid sequence alignment (Fig. 2) identifies allele C to be ancestral. Therefore, allele G is a derived but relatively ancient allele that segregates in *Ovis gmelini* and *Ovis aries*. The 167 bp insertion in the promotor region is also derived but more recent and segregates exclusively in domestic sheep and predominantly in long-tailed sheep breeds. This insertion occurred in the haplotype with the older missense allele G and both derived alleles segregate as a block. So, we did not observe the insertion without allele G, but we did observe the older allele G without insertion. The presence of the insertion in long-tailed sheep breeds and its location in the promotor region of *HOXB13* increases the probability of the insertion to be the causative variant for the long tail phenotype. Most likely because of its location the insertion modulates the promoter activity of *HOXB13* and leads to a longer tail by reducing the expression of the *HOXB13* gene. Indeed, a recent study by Li et al.^35^ also detected an insertion of 169 bp close to the 5’ UTR of *HOXB13* at position 37,525,005 (ARS-UI_Ramb_v2.0) using the graph-assembly-based method on the Pac-bio sequencing data of 13 different sheep breeds including Merino. Therefore, it is likely this study and Li et al.^35^ both identified the same insertion. The small differences in length and position of this insertion can be attributed to sequencing error due to the presence of long homopolymer of “T” base in the identified SINE repeat element. Second generation short reads sequencing technology coupled with assembly errors can make finding promising structural candidate variants difficult and thus visual examination of the targeted region should be considered. However, long-read sequencing technology like PacBio and ONT have already proven to be effective in identifying such SVs.

Interestingly, the missense mutation (*rs413316737*) in the first exon of *HOXB13* (37,290,361 C→G) is included on the OvineHD array as SNP marker *oar3_OAR11_37337253*. This offers the possibility to check the allele distribution in available open-source genotypes (Supplementary Table S5). It is noteworthy, that top-allele G of *oar3_OAR11_37337253* correspond to ancestral C in the coding sequence of the *HOXB13*. Genotyping of the candidate SNP and insertion throughout the mapping design provides the opportunity to test the efficiency of GWAS and cLDLA with the candidates as a marker and as a fixed effect. The inclusion of the candidates as a marker supports a very strong association (i.e., *P* = 6.2 × 10^−12^) or identity of the *rs413316737* and/or insertion with the causative locus. Additionally, it demonstrates the power of GWAS when the design includes causal variants or markers with population-wide LD. On the other hand, the inclusion of candidates as markers in haplotype-based mapping leads only to partial change, i.e., the mapping significance remains about the same, but the mapping peak is 9-times closer to the most plausible candidate gene. Even more conclusive is the impact of these mutations as a fixed effect in the model, because the correction for the true causal variant should cancel out the LRT peak on OAR11 and if this explains a full additive-genetic variance, the heritability should also be reduced towards zero. Indeed, this model erase the LRT peak on OAR11 (Fig. 1d) but the heritability remains relatively high (0.898). We thus gathered further evidence pointing to a missense mutation in the first exon of *HOXB13* and/or a structural variation in the promotor region of *HOXB13* as plausible causal mutations but also confirmed the presence of other, yet unknown causal variants that explain a large part of the phenotypic variance. Conversely, it is also possible that heritability estimate of the present study is inflated because of the pedigree-associated genetic variants and the shared environment^37,38^ of our samples and therefore, the variations around *HOXB13* contribute more to TL than these results suggest.

*HOXB13* belongs to the family of homeobox genes, which were first described in *Drosophila melanogaster*^46^. This gene codes for transcription factors and play an important role in structuring the body plan during embryogenesis (reviewed by Diaz-Cuadros et al.^45^). In mammals, there are 39 *Hox* genes organized in four clusters and 13 paralogous groups^47^. There is functional redundancy among the paralogous *Hox* genes^48^ and the paralog alleles can compensate for each other to a certain degree^49^. Therefore, the loss of function of one *Hox* gene from a cluster is usually compensated by the functionality of an intact paralogue, and only the loss of function of several paralogs results in more severe consequences in axial structuring^47^. There is indirect evidence that *HOXB6*^50^ and *HOXB8*^51^ may influence TL and embryonic viability. However, capture sequencing finds no genetic variants associated with TL in these two candidate genes. Therefore, these candidates are not discussed further.

As vertebrate embryos develop progressively from head to tail, the HOX13 paralogous group has been proposed to control axis termination^45^, which is mainly achieved through regulation of proliferation and apoptosis activity in the posterior embryonic regions^52^. In mice, loss-of-function mutations in *Hoxb13* lead to overgrowth of the spinal cord and caudal vertebrae in homozygous mice. These animals consequently show longer and thicker tails while viability and fertility remain unaffected^52^. Premature expression of genes of the *Hox13* paralogous group, on the other hand, negatively influences the extension of the caudal axis and results in a truncated phenotype^51^. Kingsley et al.^53^ also suggested that *Hoxd13* plays a role in the development of natural TL variation and found out that long-tailed forest mice express less *Hoxd13* in the embryonic tail bud than short-tailed prairie mice and (therefore) show better climbing performance. Among all candidate genes from the *Hox* family, *HOXB13* is thus the best functional candidate gene. Moreover, Li et al.^35^ used previously published RNA-Seq data of sheep colon, performed luciferase reporter assays, and indicated an association of lower expression of *HOXB13* with insertion in its promotor region. This is in the line with above findings in mice. However, due to complete linkage, we cannot exclude the cooperative causality of the insertion and missense mutation in exon 1. The *HOXB13* is expressed in the prostate of adult humans and is intensively studied as a candidate biomarker for the prognosis of prostate cancer (see Ouhtit et al.^54^ for review). Studies show that missense mutations in the coding sequence of *HOXB13* can change the affinity^55^ or half-life^56^ of heterodimer between HOXB13 and, e.g., MEIS1 proteins. However, it was not within the scope of this study to investigate the affinity between different transcription factors in growing sheep embryos or cell lines. It is well known that the SINE insertion alone can alter gene expression in multiple ways^57^, but we propose to test the hypothesis of a possible causality of the combination of altered expression and altered amino acid sequence.

As already introduced, attempts were made in 1970s and 1990s to breed short-tailed Romney^19^ and Merino^12,15^ sheep. These attempts failed due to evidence of reduced viability of presumably homozygous short-tailed Romney embryos and by increased incidences of rear-end defects in short-tailed Merinos. However, these observations contradict the fact that the short tail is the ancestral trait and that old Nordic short-tailed breeds like Romanov and Finnsheep are very viable and highly fertile. Therefore, we assume that these early experiments were not carried out with animals that had shorter tails due to ancestral genetic variants, but due to some recent deleterious variants. James et al.^15^ mentioned that the TLs of the four sires measured before mating were all less than 5 cm. This is very short for an adult ram, even much shorter than the tail of short-tailed adult Romanov rams (~20 cm). Also, Romney sheep used for experimental mating by Carter^19^ were described as “tailless”. Therefore, short or tailless phenotypes described in initial sheep breeding attempts^15,19^ are comparable with deleterious mutations described for certain dog and cat breeds^22–24,42,43^ rather than to some ancestral alleles. The causal candidates for short tails detected in this study are highly frequent or fixed in some very viable and highly fertile breeds (e.g., Finnsheep, Romanov, Dalapaels, and Soay, Supplementary Table S5). Therefore, the selection toward the ancestral allele do not carry any detrimental side effects for fertility or malformations^58^ and can be achieved without introgression. However, further studies would be necessary to detect the causality between the extremely short-tailed animals and the associated undesirable phenotypes as reported previously^15,19,22–24,42,43^.

In our design, all homozygous ancestral and the majority (79.7%) of heterozygous lambs could be classified as short-tailed, while homozygous derived lambs are mostly (71.4%) but not always long-tailed (Table 4). This could be caused by interaction (epistasis) with alleles at other loci in the sheep genome or simply by the polygenic nature of the high proportion of additive-genetic variance which was still not explained by the mutations discovered here (0.898). In addition, functional redundancy^48^ and synergistic interaction^59^ between the paralogus HOX genes could contribute to the additional complexity of the phenotype. However, the genomic regions containing the HOXA (OAR4, 68 Mb), HOXC (OAR3, 132 Mb), and HOXD (OAR2, 132 Mb) gene clusters show no signals in the cLDLA analyses without (Model 3**)** and with (Model 5) candidate mutations as a fixed effect (Fig. 1c, d). Tacking together, to map some remaining causal variants in long-tailed breeds, we will need a design with much higher statistical power than the one carried out in the present study. The synergistic interaction of different paralogous HOX genes could also cause a possible link between TL and fine wool in sheep, since it is known that *HOXB13*^60^ and *HOXC13*^61^ has an impact in hair follicle morphogenesis and therefore controls hair formation. This would explain an already discussed association between TL and fine wool. Further studies are needed to prove or reject a correlation by taking into account the expression patterns of the different *HOX13* genes.

Our results provide a comprehensive insight into the genetic variance of TL in long-tailed Merino sheep and offer information towards direct gene-assisted selection for shorter tails and thus contribute to animal welfare by avoiding tail docking in the future. Part of the society, which is actively committed to animal welfare, frequently has prejudices against genetic methods in general. Therefore, it should be pointed out once again that this is a natural original genetic variant and that selection in favor of this variant serves to restore the most natural original trait. We would not call it a “repair”, but a “back to the roots”. Additional to commercial and animal welfare aspects this and follow-up studies could contribute to a better understanding of embryonal development too. According to Aires et al.^62^ quantitative differences within the *Gdf11-Lin28-Hoxb13-Hoxc13* gene-network might account for the tail size variability observed among vertebrate species. Thereby, the determination of the TL could result from the relative intensity or the sequence of the individual network components. The mechanisms regulating tail size are still not fully understood especially the pathways downstream of *Lin28* and *Hox13* genes in the *Gdf11-Lin28-Hoxb13-Hoxc13* network. Therefore, Aires et al.^62^ suggested testing the gene-network parameters in embryos of vertebrate species with different tail sizes. The embryos of a sheep breed with an ancestral short tail and a derived long tail are suitable candidates for studying the control of axis termination. In the meantime, the mapping design could be extended and improved to map genes pointing to further network candidates, possibly downstream mediators of the *HOXB13*.

In conclusion, we revealed a SNP in the first Exon of *HOXB13* and a SV in the promotor region of *HOXB13* on OAR11, which are highly associated with TL in the Merinolandschaf breed. The results presented here suggest that the TL depends on many loci with minor effects, whereas mentioned variations around *HOXB13* cause the main effect in TL. This is evident from the only slight reduction in heritability after correcting for these most effective variations. To detect the additional causal loci a more powerful design is needed. Finally, we want to stress that selection for shorter tails is possible without introgression and without negative side effects and that this selection is ethically unproblematic as it leads to an increased frequency of the ancestral allele

## Methods

### Animal samples and phenotypes

**Fig. 4:**
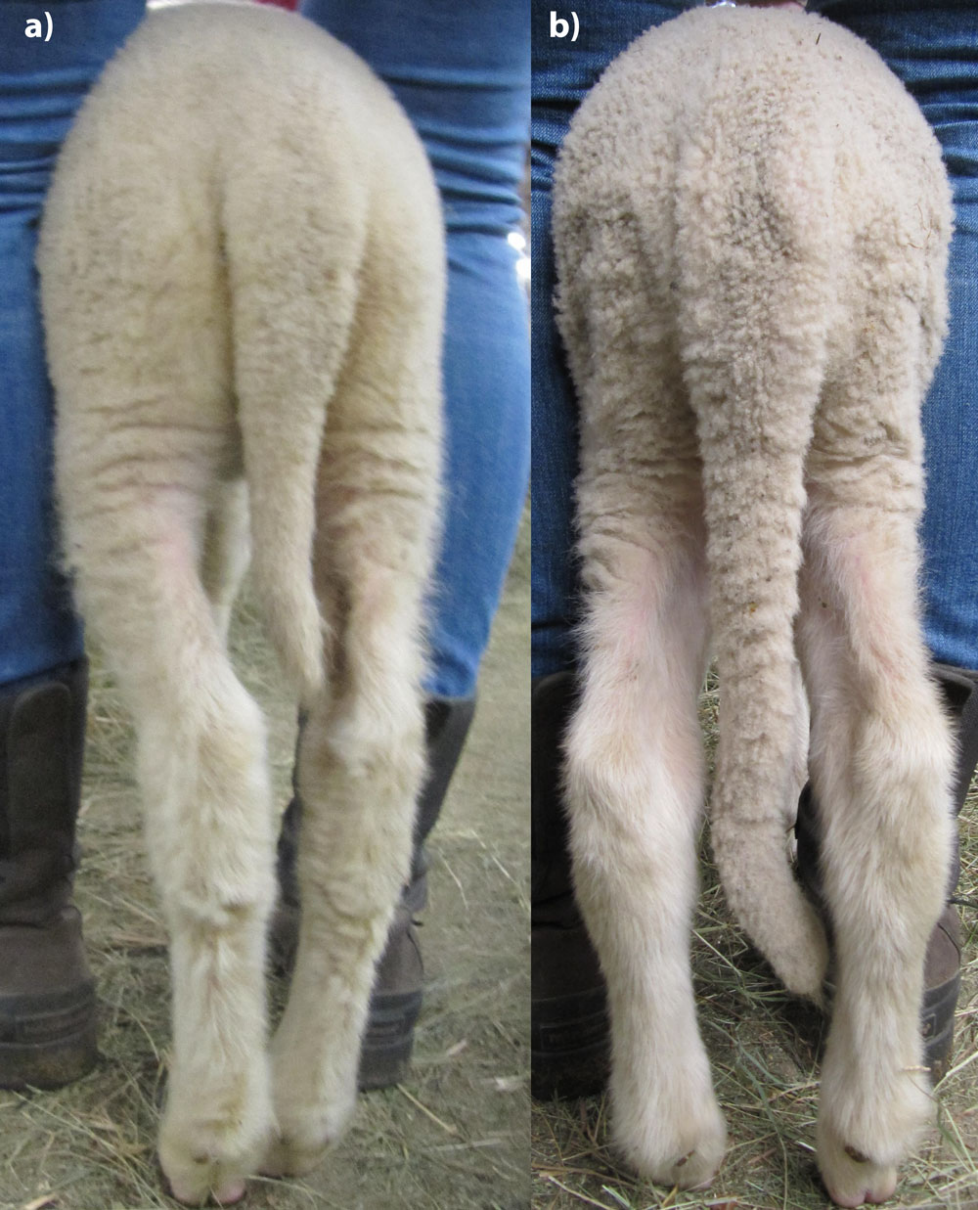
Natural tail length variation in Merinolandschaf. Photo of a short-tailed (**a**) and a long-tailed (**b**) lamb, both lambs were ~5 weeks old and not docked.

The entire mapping design of 362 phenotyped and genotyped animals were collected in three phases: (i) 236 Merinolandschaf lambs with very short (104) or long (132) tails were selected for phenotyping and sampling from 2293 visually inspected lambs on a farm in Lower Bavaria with no custom of tail docking, (ii) 102 random male lambs from the same farm were phenotyped and sampled, i.e., without pre-selection by visual inspection of tail-length and (iii) 24 Merinolandschaf lambs with short (19) or long (5) tails were selected from 102 visually inspected lambs from the sheep flock of the teaching and research farm “Oberer Hardthof” at the Justus Liebig University of Giessen (JLU). Figure 4 shows the variation in the tail phenotype in Merinolandschaf and Supplementary Fig. S5 shows the distribution of TL from the 260 out of 2395 lambs that were selected for sampling after visual inspection. Phenotyping of these 362 lambs was conducted according to the method proposed by Eck et al.^63^. Although an age of 5 weeks proved to be the optimal time point for phenotyping, we sampled and phenotyped also younger and older lambs (Supplementary Table S6 shows descriptive statistics about the age and other phenotypic traits) in order not to disturb the work processes on the farm. TL was measured with a custom-made wooden board from the anus to the tip of the tail, BW with a standard scale, and WH with a metal measuring device from dog sports from the floor to the highest point of the withers. Furthermore, gender, age, and litter size were recorded. Unfortunately, the age and litter size are only approximately known for randomly sampled 102 animals. To improve haplotype inference, we sampled and genotyped 22 putative fathers of the above lambs. These rams were not phenotyped and contributed only indirectly to the QTL mapping.

The collection of blood samples for this study was approved by the ethics committee of the Veterinary Faculty of LMU Munich. All blood samples were taken by experienced and qualified veterinarians and under a permit from the Government of Upper Bavaria (permit number: 55.2-1-54-2532.0-47-2016), or the Regional Council of Gießen, Hassia (KTV number: 19 c 20 15 h 02 Gi 19/1 KTV 22/2020).

### Genotypes

All 362 phenotyped lambs and 22 sires were genotyped using Illumina’s OvineSNP50 BeadChip (Illumina, San Diego, USA) according to the manufacturer’s specifications. All physical marker positions were determined on the ovine reference genome assembly Oar_4.0 (https://www.ncbi.nlm.nih.gov/assembly/GCF_000298735.2) and the positions of all markers or sequences in this work correspond to the Oar_4.0 reference genome unless otherwise stated. The chip contains 54,241 SNPs almost evenly distributed across the genome with an average marker spacing of 50.9 Kb^64^. Not all of these 54,241 SNPs were used for mapping. We filtered SNPs according to the following exclusion criteria: (i) unsuccessful genotyping in >5% of the animals, (ii) frequent paternity conflicts in animals with known paternity, (iii) unknown position in the reference genome, (iv) minor allele frequency (MAF) of >0.025 and (v) localization on a sex chromosome since the analyses were exclusively carried out on autosomes. As a result, 45,114 markers remained in the marker set for the mapping analyses. Haplotype phasing and imputation were conducted using a Hidden Markov Model implemented in *Beagle* version 5.0^65^. To improve the accuracy of haplotyping and imputation, genotype and pedigree information from ~5100 additional animals genotyped with the OvineSNP50 BeadChip but not phenotyped were added to the haplotyping design^66^.

### Estimation of heritability and mapping

First, we tested the heritability of the TL in the pure Merinolandschaf breed. For this purpose we used the software *GCTA* v1.93.2, which has been extended with GRM, a tool for estimating the genetic relationship matrix and a genomic-relatedness-based restricted maximum-likelihood approach (GREML), to estimate the proportion of variance explained by all SNPs (the SNP-based heritability)^67^. The sex, age, BW, and WH of the lambs at phenotyping were modeled as fixed effects.

### Mixed linear model-based association analysis (MLMA)

To map a putative TL locus, we performed MLMA analyses with a leave-one-chromosome-out approach as implemented in the software *GCTA* v1.93.2^68^. The model is described below:

1 $${{{{{\bf{y}}}}}}={{{{{\bf{X}}}}}}\ss+{{{{{{\bf{Z}}}}}}}_{{{{{{\bf{a}}}}}}}{{{{{\bf{a}}}}}}+{{{{{{\bf{Z}}}}}}}_{{{{{{\bf{u}}}}}}}{{{{{\bf{u}}}}}}+{{{{{\bf{e}}}}}}$$

where **y** is the vector of TLs (cm), **ß** is a vector of fixed effects including the mean, sex, age, BW in kg, and WH in cm at tail phenotyping (BW and WH data were both standardized and centered), **a** is the vector of the additive effect (fixed) of the candidate SNP to be tested for association, **u** is the vector of polygenic effect (random or accumulated) of all markers, excluding those on the chromosome which contains the candidate SNP and **e** is a vector of residuals. **X**, **Z**_**a**_, and **Z**_**u**_ are the incidence matrices for **ß**, **a**, and **u**, respectively.

The suggestive significance threshold was set at *P* < 1/*N* and the genome-wide significance threshold at *P* < 0.05/*N*, according to Bonferroni method, *N* stands for the number of markers^69^. For the initial MLMA-analysis (Model 1) we considered 45,114 markers resulting in the suggestive *P* value at 2.22 × 10^−5^ and genome-wide at 1.11 × 10^−6^.

A second MLMA (Model 2) analysis included one additional locus, which we detected during this research, as a consequence, we considered 45,115 markers. The suggestive and genome-wide significance threshold remained the same.

### Combined linkage disequilibrium and linkage analysis (cLDLA)

Parallel with SNP-based association analyses using MLMA, we performed multiple haplotype-based cLDLA analyses^70^, which have been successfully used for binary and quantitative trait mapping in previous studies^71–74^.

To correct for familial relationships and population stratification, unified additive relationships (UARs) were estimated between all animals on a genome-wide level^75^. The inverse of the UAR matrix was then included in the variance component analysis. To account for linkage disequilibrium in the form of local haplotype relationships, the Locus IBD (LocIBD) was estimated according to the method of Meuwissen and Goddard^76^ using sliding windows of 40 SNPs. For each window, we estimated LocIBD in the middle, i.e., between SNPs 20 and 21, based on the flanking marker haplotypes. Following the method for additive-genetic relationship matrices by Lee and Van der Werf^77^, the matrix of LocIBD-values was converted into a diplotype relationship matrix (**D**_**RM**_).

Variance component analyses were carried out with the program *ASReml*^25^ according to the method of Meuwissen et al.^70^. *ASReml* estimated the maximum likelihood, variance components and fixed and random effects simultaneously by considering the genome-wide UAR matrix as well as the locus-specific (**D**_**RM**_) relationships matrices in the following mixed linear model:

2 $${{{{{\rm{y}}}}}}={{{{{\rm{X}}}}}}{{\ss}}+{{{{{\rm{Z}}}}}}_1{{{{{\rm{u}}}}}}+{{{{{\rm{Z}}}}}}_2{{{{{\rm{q}}}}}}+e$$

where **y** is again the vector of TLs and **β** is the vector of fixed effects (BW, WH, sex, age, and the overall mean µ; BW and WH data were both standardized and centered). The vector **u** is the vector of random polygenic effects (with **u**~*N*(0, ${{{{{{\bf{G}}}}}}\sigma }_{u}^{2}$) where **G** represents the matrix of genome-wide UAR estimations), **q** is the vector of random additive-genetic QTL effects (with **q**~*N*(0,${{{{{{\bf{D}}}}}}}_{{{{{{\bf{RMp}}}}}}}{\sigma }_{q}^{2}$) where **D**_**RMp**_ is the diplotype relationship matrix at position *p* of a supposed QTL) and **e** is the vector of random residual effects (with **e**~*N*(0, ${{{{{{\bf{I}}}}}}\sigma }_{e}^{2}$), where **I** is an identity matrix). It is assumed that **u**, **q**, and **e** are not correlated. **X, Z**_**1**_, and **Z**_**2**_ are incidence matrices linking the observed values with the fixed and random QTL effects.

The variance components and likelihood estimated with *ASReml* were then used in a likelihood ratio test statistic (*LRT*). The *LRT* values follow a *χ*^2^ distribution with one degree of freedom^78^ and were calculated as:

3 $${{{{{\boldsymbol{LRT}}}}}}{{{{{\boldsymbol{p}}}}}}=-{{{{{\bf{2}}}}}}\times ({{{{{\bf{log }}}}}}({{{{{\boldsymbol{L}}}}}}{{{{{\bf{0}}}}}})-{{{{{\mathbf{log }}}}}}({{{{{\boldsymbol{L}}}}}}{{{{{\bf{1}}}}}}{{{{{\boldsymbol{p}}}}}}))$$

where log(*L*_*0*_) is the logarithm of the likelihood estimated by *ASReml* for the model without QTL effects and log(*L*_*1p*_) the model with QTL effects at the center of the window *p*.

To obtain a significance threshold, a Bonferroni correction was carried out to account for multiple testing due to the 40-SNP sliding windows^79^. This resulted in a corrected *P* value of <4.44× 10^−5^ (i.e., 0.05/1127 where 1127 is the number of non-overlapping 40-SNP windows), and a corresponding *LRT* value with genome-wide significance is equal to 16.67.

In addition to Model 3 described above, we performed two further genome-wide cLDLA analyses, with both analyses using candidate locus genotypes for the same set of animals. Model 4 contains only one additional locus and is therefore comparable to Model 2 of MLMA. In Model 5, the genotypes of the same candidate locus are considered as a fixed effect, i.e., **β** is the vector of µ, sex, age, BW, WH, and candidate locus effects. A comprehensive overview of the different models is provided in Table 1. For all maxima of the LRT curve (*LRT*max) that exceeded the genome-wide significance threshold, the 2-LOD (logarithm of the odds) criterion was used to determine the associated CIs^80^. Closely located LRT peaks were assumed to belong to the same QTL as described by Müller et al.^74^.

The results of the analyses were presented as Manhatten plots produced by the *R* package *qqman*^81^.

### Annotation of gene content and gene set enrichment analysis

The CIs of the *LRT*max were compared with a map of annotated genes in the UCSC Genome Browser Oar_v4.0/oviAri4 Assembly (https://genome.ucsc.edu/cgi-bin/hgGateway) using the “RefSeq Genes” track. We refer to the assembly of Oar_v3.1 (https://www.ncbi.nlm.nih.gov/assembly/GCF_000298735.1), the *Mus musculus* Assembly (https://www.ncbi.nlm.nih.gov/assembly/GCF_000001635.27) and the *Homo sapiens* Assembly (https://www.ncbi.nlm.nih.gov/assembly/GCF_000001405.39) as well to consider genes encompassing the CIs. Next, gene set enrichment analyses were carried out with the software *Enrichr* (Ontologies, MGI Mammalian Phenotype Level 4 2019)^82,83^.

### DNA extraction, sequencing, and analysis of the sequences

Genomic regions where the LRT curve reached the maximum values above the genome-wide significance threshold (in Model 3) were sequenced using targeted capture sequencing on 48 selected lambs (23 long-tailed and 25 short-tailed). To minimize the risk of missing some causal variations that are close but outside the CI, we increased the candidate regions on both sides by ~300 Kb. This resulted in the captured regions on the sheep chromosome 2 (OAR2) from 93,200,000 to 96,700,000 bp and on chromosome 11 (OAR11) from 36,600,000 to 37,900,000 bp. Genomic DNA was extracted from the blood samples using the ReliaPrep™ Blood gDNA Miniprep System.

WGS libraries were prepared from 250 ng of genomic DNA by tagmentation with the NexteraFlex kit from Illumina. Subsequently, the libraries were dual-barcoded and amplified by PCR, purified with SPRI beads and pooled in equimolar amounts. The pooled libraries were enriched for the target regions by hybridization to an Agilent capture array with 244k oligo spots. The oligo probes were selected from the repeat-masked DNA sequence as all possible 60mers that do not overlap with repeat-masked bases and that are staggered in 15 nt tiling steps concerning their neighbors. After 65 hours of hybridization in the presence of cot-I sheep DNA and adapter blocking oligos at 65 °C the capture array was washed and the captured library molecules were eluted at 95 °C for 10 min in a volume of 500 µl DNA-grade water. The enriched libraries were then amplified by PCR, analyzed on the Bioanalyzer and sequenced in 2 × 110 bp paired-end mode on a P2 flowcell of a NextSeq1000 sequencer from Illumina.

*Sickle*^84^ was used to trim the adaptors and filter low-quality sequences of the raw reads in the FASTQ files. With *FastQC*^85^, we assessed the quality parameters of the filtered sequencing data. The filtered reads were mapped to the sheep reference genome OAR_v4.0 using the default parameters of *BWA-MEM*^86^ alignment tool. To convert the SAM files into coordinate sorted BAM files and to remove the duplicated reads, *Samtools*^87^ and *Picard*^88^ were used. Base quality recalibration and indel realignment were done with *GATK*^27^. SNPs and small indels calling was performed with *GATK HaplotypeCaller*^27^ using: (i) 37 wild sheep genomes, (ii) 16 domestic sheep genomes (representing 8 samples of short-tailed breeds and long-tailed breeds each) and (iii) two sequence pools, representing short and long-tailed group of the 48 Merinolandschaf lambs. These 48 lambs were sequenced individually (see above), then reads were pooled to increase the sequencing coverage of both groups.

The wild and domestic sheep genomes downloaded from NCBI sequence read archive are shown in Supplementary Table S4. To filter the obtained *GATK* short variants, the criteria described in the Supplementary methods were applied. To detect structural variants (SV) we used *SMOOVE* (*LUMPY*)^32^ and *DELLY*^33^ with default parameters in the pooled reads of short- and long-tailed lambs.

To ensure that we do not miss any candidate variant, we performed a visual examination of the captured regions using *JBrowse*^28^, focusing on the region near the candidate gene detected here.

### Validation of candidate SNP using PCR

For one detected candidate SNP, we performed genotyping by PCR-RFLP and electrophoresis on a 2% agarose gel on all 362 sampled lambs, and there 17 confirmed fathers. The PCR primer sequences designed by *Primer3*^89^ were TTTAAAACGCTTTGGATT (forward) and CACTCGGCAGGAGTAGTA (reverse). The used restriction enzyme was *BsrI*. It recognizes the mutant sequence TGAC/CN, where the G is the variable base and the “/” presents the site where cutting is performed. DNA amplification was performed with 35 cycles. The total reaction mixture was 15.0 µl containing 3.0 µl 5× buffer, 1.5 µl dNTPs (10 mM), 0.6 µl of 10 µM forward and reverse primers, respectively, 1.0 µl DNA (15 ng/µl), 0.07 µl GoTaq®G2 DNA Polymerase (Promega, Madison, Wisconsin, USA) and distilled water.

We used 1.5 U of the enzyme *BsrI*, 3.0 µl DNA (PCR product), 2 µl Cut Smart Buffer, and distilled water for a total reaction volume of 20 µl. The reaction mixture was afterward incubated for 3 h at 65 °C. In the final step, we separated the DNA fragments by size and visualized them by GelRed™-stained agarose gel electrophoresis. Only sequences harboring the derived allele (SNP G) were cut, the two resulting fragments had a length of 120 bp and 259 bp. The sequence with the ancestral allele (SNP C) retained its length of 379 bp.

### Validation of candidate insertion using PCR and Sanger sequencing

We also performed genotyping by PCR and electrophoresis on a 2% agarose gel on the same 379 sampled sheep for one detected causal candidate insertion. Multiple PCR primer sequences designed by *Primer3*^89^ were tested (see Supplementary Table S7), those that worked best are TTTATGAGCTTCTCTCCGCCA (forward) and CACTCGGCAGGAGTAGTA (reverse). DNA amplification was performed with 35 cycles. The total reaction mixture was 25.0 µl containing 5.0 µl 5× buffer, 2.5 µl dNTPs (10 mM), 1 µl of 10 µM forward and reverse Primer, respectively, 1.0 µl DNA (15 ng/µl), 0.07 µl GoTaq®G2 DNA Polymerase (Promega, Madison, Wisconsin, USA) and distilled water. In the final step, we separated the amplicons by size and visualized them by GelRed™-stained agarose gel electrophoresis.

Two lambs, which are homozygous for the SV, one lamb, which is homozygous for the ancestral allele, and two heterozygous lambs were resequenced using Sanger sequencing with the above-mentioned best working primers. The amplicons were sequenced using the cycle sequencing technology (dideoxy chain termination/cycle sequencing) on ABI 3730XL sequencing machines (Eurofins Genomics, Germany). The sequenced data were analyzed using *SnapGene* software (from Insightful Science; available at https://www.snapgene.com/).

### Reporting summary

Further information on research design is available in the Nature Research Reporting Summary linked to this article.

## Supplementary information

Supplementary Information

Reporting Summary

## Data Availability

Genotype and phenotype data as well as Sanger sequences that support the findings of this study have been deposited in Figshare (10.6084/m9.figshare.19375712, 10.6084/m9.figshare.19368962, 10.6084/m9.figshare.19368905, 10.6084/m9.figshare.19368734). Also, the source data for the plots (results of the MLMAs and the cLDLAs) have been deposited on Figshare (10.6084/m9.figshare.20439849 and 10.6084/m9.figshare.20439582). Capture sequencing data have been deposited in European Nucleotide Archive (ENA) with the study accession PRJEB51698. All other data are available from the corresponding author on reasonable request.
