# Supplementary material for: Fine-mapping and identification of candidate causal genes for tail length in the Merinolandschaf breed
Source: Commun Biol. 2022 Sep 6;5:918. doi: 10.1038/s42003-022-03854-3 (PMC9448734; doi:10.1038/s42003-022-03854-3)
Supplement: Supplementary file 1 — Supplementary Information [file 42003_2022_3854_MOESM1_ESM.pdf]

Fine-mapping and identification of candidate causal genes for tail length in the Merinolandschaf breed

Dominik Karl Lagler[1,2], Elisabeth Hannemann[1], Kim Eck[1,2], Jürgen Klawatsch[1,2], Doris Seichter[2], Ingolf Russ[2], Christian Mendel[3], Gesine Lühken[4], Stefan Krebs[5], Helmut Blum[5], Maulik Upadhyay[1], Ivica Međugorac[1*]

[1] Population Genomics Group, Department of Veterinary Sciences, LMU Munich, Lena-Christ-Str. 48, 82152 Martinsried, Germany

[2] Tierzuchtforschung e.V. München, Senator-Gerauer-Str. 23, 85586 Poing, Germany

[3] Institute for Animal Breeding, Bavarian State Research Center for Agriculture, Prof.-Dürrwaechter-Platz 1, 85586 Poing, Germany

[4] Department of Animal Breeding and Genetics, JLU Gießen, Ludwigstr. 21a, 35390 Gießen, Germany

[5] Laboratory for Functional Genome Analysis, Gene Center, Ludwig-Maximilians-University Munich, 80539 Munich, Germany

* Corresponding author: Dr. Ivica Međugorac (i.medugorac@lmu.de)

Supplementary Information

Supplementary Figures

a)

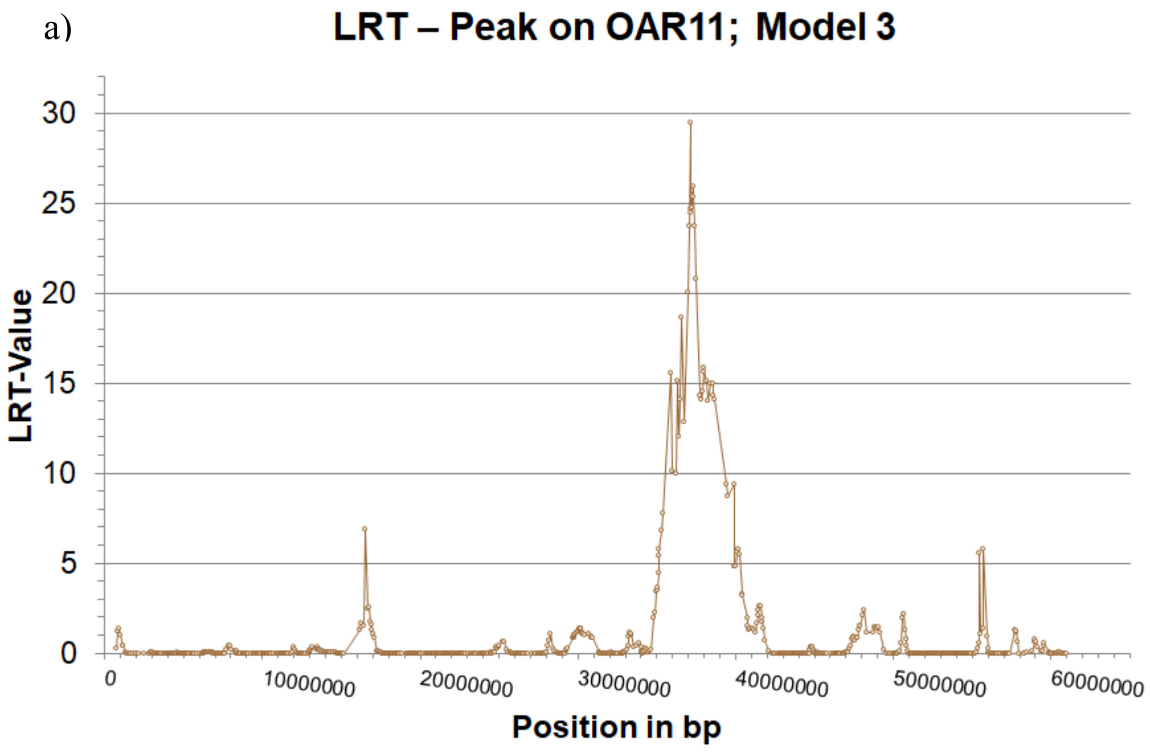

b)

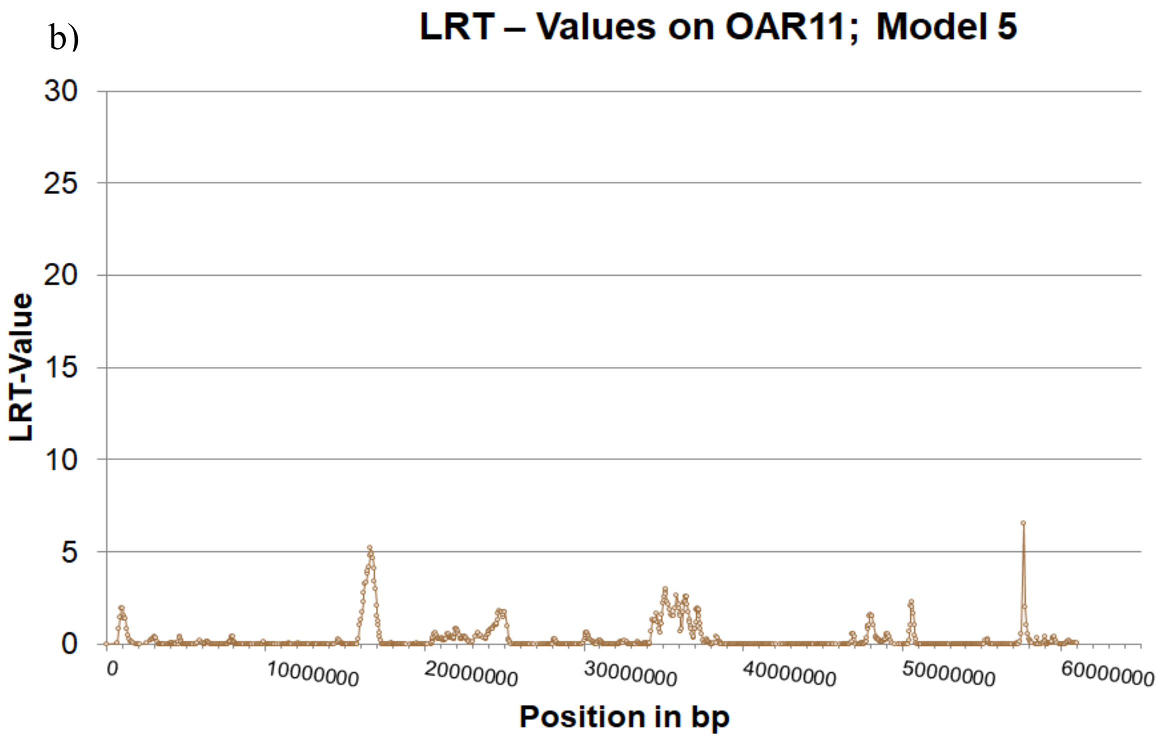

Supplementary Figure S1: LRT-Peaks on OAR11. a) shows the peak for Model 3 and b) for Model 5

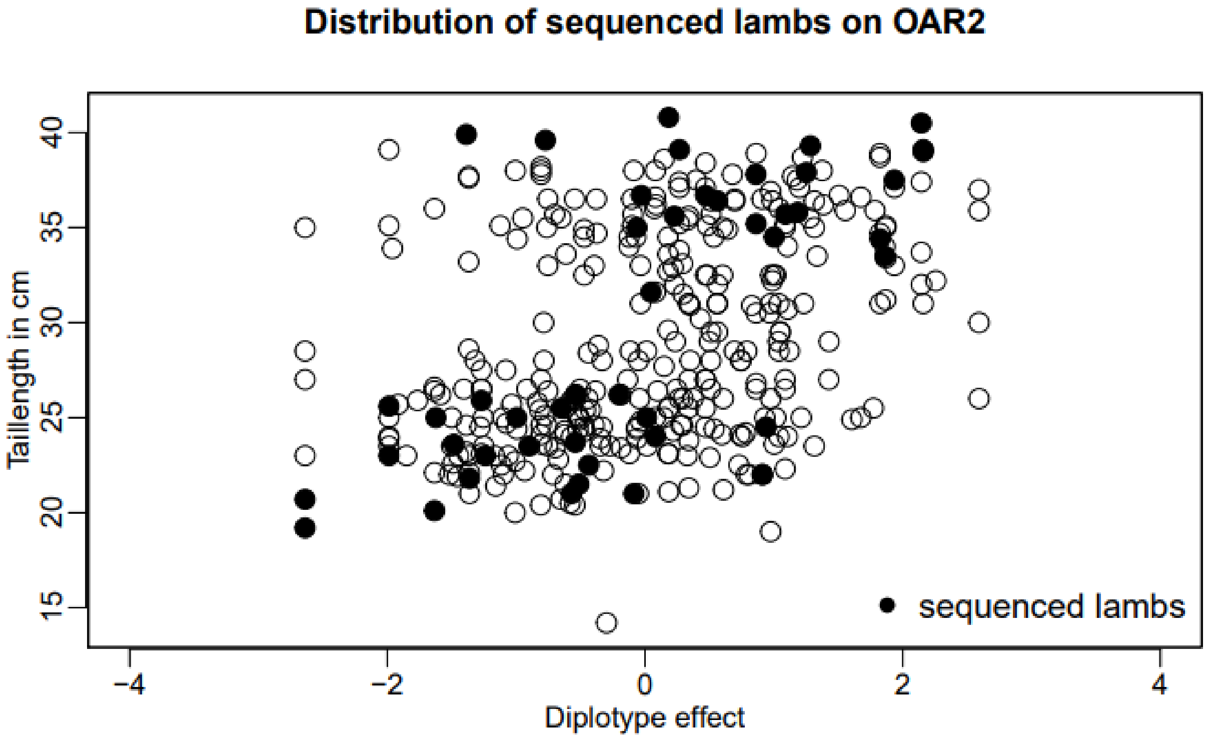

Supplementary Figure S2: Each dot presents one of the 362 investigated lambs. Sequenced individuals are shown as • and were selected based on their age, tail length and diplotype effect on OAR11 (where the peak occurred). The figure indicates no association between the tail length and the diplotype effect on OAR2, because both the types of animals, animals with low diplotype effect and animals with high diplotype effect, have long as well as short tails.

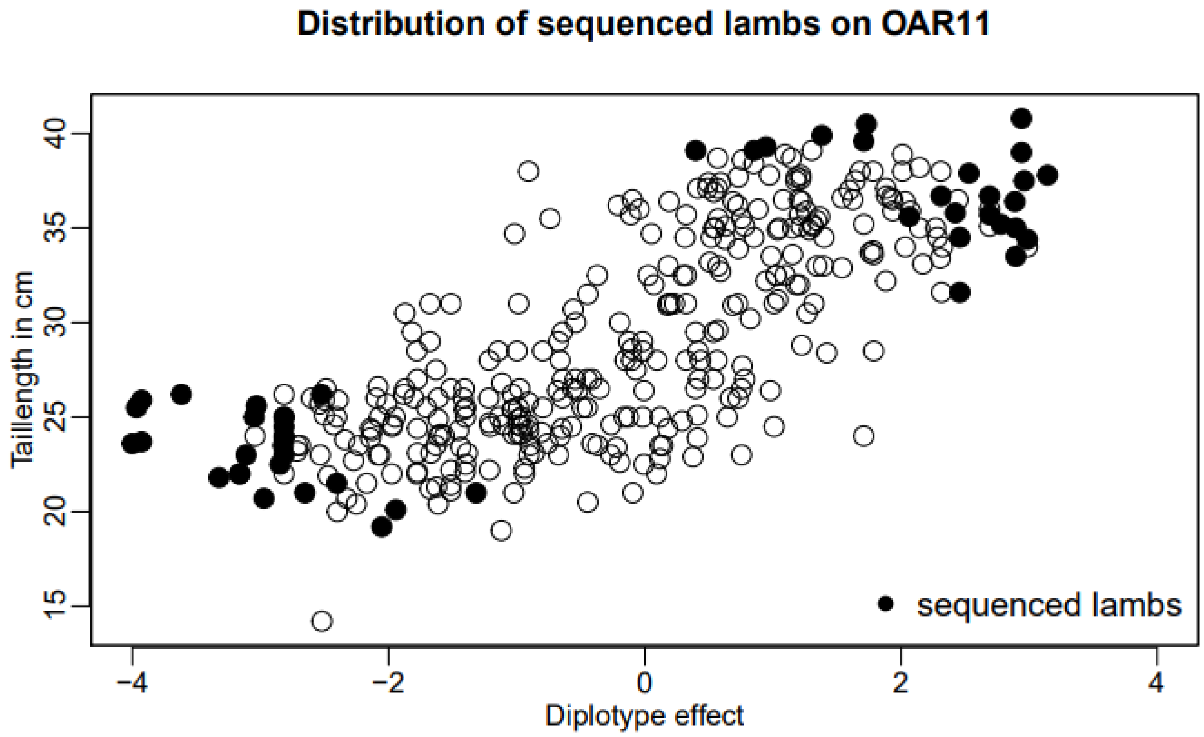

Supplementary Figure S3: Each dot presents one of the 362 investigated lambs. Sequenced individuals are shwon as • and were selected based on their age, tail length and diplotype effect on OAR11 (where the peak occurred). The graphic shows the association between tail length and the diplotype effect on OAR11.

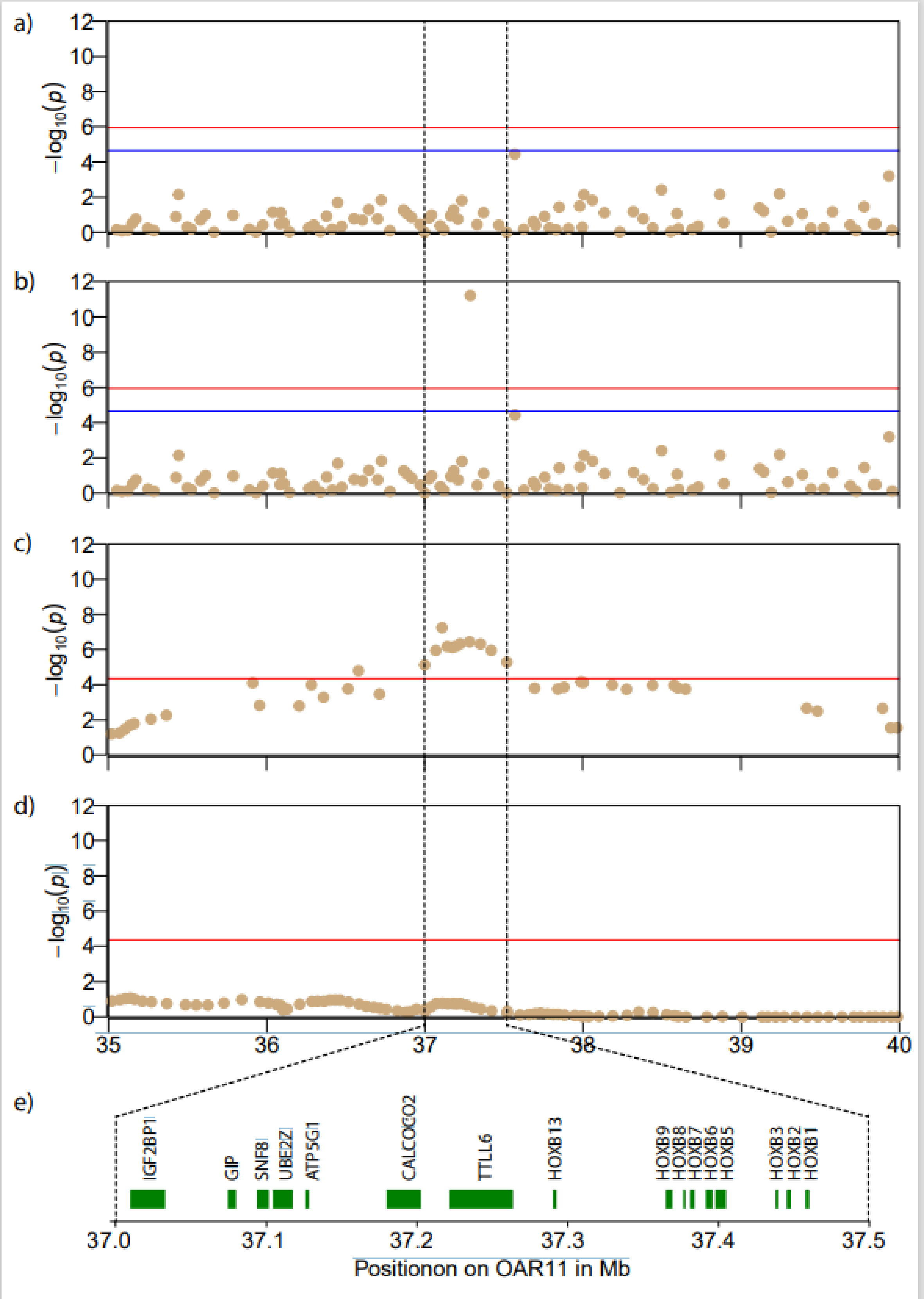

Supplementary Figure S4: LocusZoom plots for the region between 35 Mb and 40 Mb on OAR 11. Panel a) shows the region for Model 1, b) for Model 2, c) for Model 3 and d) for Model 5. Panel e) shows the annotation of the genes within the confidence interval (between positions 37,000,925 bp and 37,521,490 bp).

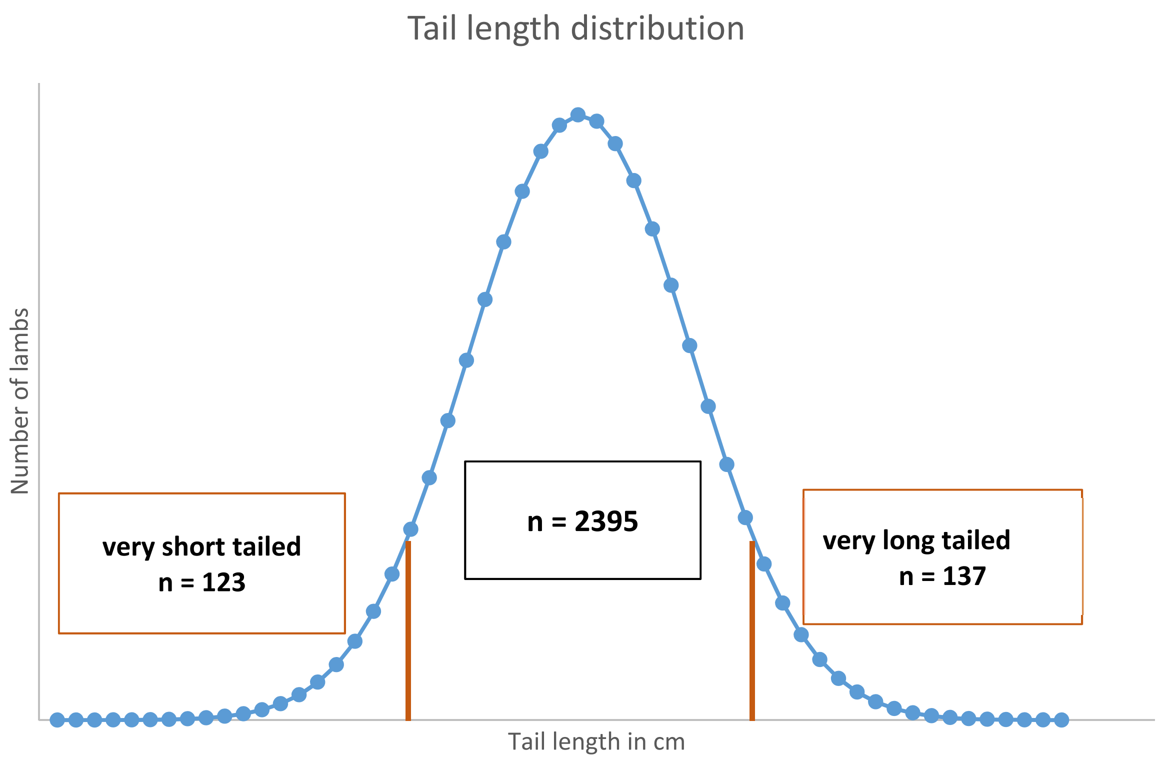

Supplementary Figure S5: Of 2395 visually inspected lambs, 260 lambs from both ends of the phenotypic spectrum (123 particularly short tailed lambs and 137 particularly long tailed lambs) were selected for genotyping with Illumina's OvineSNP50 BeadChip, these 260 lambs show a bimodal distribution. 102 additional lambs were randomly selected (without visual pre-selection) and therefore show a normal distribution.

Supplementary Tables

Supplementary Table S1: Regression analysis for OAR2, where in the column (1) diplotype effect (DipE) is the independent variable and in column (2) DipE, age, sex body weight (BodyW) and withers height (WitherH) are the independent variable. In both models, tail length (TailL) is the dependent variable.

	Dependent variable: TailL	
	(1)	(2)
DipE	$2{,}038^{***}$ (0,252)	$2{,}047^{***}$ (0,204)
Age		$-0{,}078^{**}$ (0,036)
Sex		-0,755 (0,472)
BodyW		$0{,}723^{***}$ (0,102)
WitherH		$0{,}212^{**}$ (0,105)
Constant	$29{,}046^{***}$ (0,275)	$11{,}816^{***}$ (3,987)
Observations	362	362
R^2	0,153	0,461
Adjusted R^2	0,151	0,454
Residual Std. Error	5,232 (df = 360)	4,197 (df = 356)
F Statistic	$65{,}213^{***}$ (df = 1; 360)	$60{,}959^{***}$ (df = 5; 356)
Note:		*p<0,1; **p<0,05; ***p<0,01

Supplementary Table S2: Regression analysis for OAR11, where in the column (1) diplotype effect (DipE) is the independent variable and in column (2) DipE, age, sex body weight (BodyW) and withers height (WitherH) are the independent variable. In both models, tail length (TailL) is the dependent variable

	Dependent variable: TailL	
	(1)	(2)
DipE	$2{,}661^{***}$(0,120)	$2{,}447^{***}$(0,087)
Age		$-0{,}077^{***}$(0,022)
Sex		$-0{,}993^{***}$(0,298)
BodyW		$0{,}621^{***}$(0,064)
WitherH		$0{,}164^{**}$(0,066)
Constant	$29{,}665^{***}$(0,196)	$16{,}452^{***}$(2,524)
Observations	362	362
R^2	0,577	0,785
Adjusted R^2	0,576	0,782
Residual Std. Error	3,698 (df = 360)	2,649 (df = 356)
F Statistic	$491{,}510^{***}$ (df = 1; 360)	$260{,}561^{***}$ (df = 5; 356)
Note:		*p<0,1; **p<0,05; ***p<0,01

Supplementary Table S3: Positions of remaining short variants, mainly SNPs, after applying filter criterions described in Supplementary Methods. Column 2 until 4 show number of short tailed and long tailed lambs with reference allele and alternative alleles (ST indis ref: Number of short tailed Merinolandschaf lambs with homozygous reference genotype; ST indis var: Number of short tailed Merinolandschaf lambs with either heterozygous or homozygous alternative genotype; LT indis ref: Number of long tailed Merinolandschaf lambs with homozygous reference genotype; LT indis var: Number of long tailed Merinolandschaf lambs with either heterozygous or homozygous alternative genotype. Also Variant IDs and the results of the Ensembl Variant Effect Predictor[1] are shown. The meaning of the variant consequence and of the impact are described on https://www.ensembl.org/Help/Glossary?id=535

Position	ST indis ref	ST indis var	LT indis ref	LT indis var	Variant ID	Consequence	Impact	SIFT
36921089	3	5	10	2	rs420125730	intron_variant (B4GALNT2)	MODIFIER	-
37079829	10	7	3	13	rs426813381	downstream_gene_variant (GIP)	MODIFIER	-
37081200	7	1	1	7	rs413378768	downstream_gene_variant (GIP)	MODIFIER	-
37081999	19	1	7	8	rs421540535	downstream_gene_variant (GIP)	MODIFIER	-
37082002	18	1	7	7	rs399930658	downstream_gene_variant (GIP)	MODIFIER	-
37274710	7	7	15	1	rs406224770	intergenic_variant (TTLL6-HOXB13)	MODIFIER	-
37275045	14	9	0	18	rs424357928	intergenic_variant (TTLL6-HOXB13)	MODIFIER	-
37275989	14	8	0	18	rs407002893	intergenic_variant (TTLL6-HOXB13)	MODIFIER	-
37277182	16	6	0	18	rs410624664	intergenic_variant (TTLL6-HOXB13)	MODIFIER	-
37278058	15	7	0	19	rs429611961	intergenic_variant (TTLL6-HOXB13)	MODIFIER	-
37278438	5	19	21	1	rs419262514	intergenic_variant (TTLL6-HOXB13)	MODIFIER	-
37278757	15	8	0	19	rs398973702	intergenic_variant (TTLL6-HOXB13)	MODIFIER	-
37278805	15	7	0	17	rs414342629	intergenic_variant (TTLL6-HOXB13)	MODIFIER	-
37279115	15	7	0	21	rs405338697	intergenic_variant (TTLL6-HOXB13)	MODIFIER	-
37279536	16	5	0	19	rs430325358	intergenic_variant (TTLL6-HOXB13)	MODIFIER	-
37279822	11	2	1	7	rs421532300	intergenic_variant (TTLL6-HOXB13)	MODIFIER	-
37285884	13	7	0	17	rs414085759	upstream_gene_variant (HOXB13)	MODIFIER	-
37288535	12	9	0	17	rs413678645	upstream_gene_variant (HOXB13)	MODIFIER	-
37290361	17	2	0	15	rs413316737	missense_variant (HOXB13)	MODERATE	0.54

Supplementary Table S4: Run numbers and bioSample ID of different sheep breeds and their genotype for the structural Variation (SV) and the SNP. Genotypes are homozygous ancestral (A/A), homozygous derived (D/D) and heterozygous (A/D)

	Sample		Genotype		Submitted by
Breed	Run number	BioSample ID	SV	SNP	
Asiatic Mouflon	ERR157938	SAMEA2012637	A/A	A/A	Genoscope *
Asiatic Mouflon	ERR157930	SAMEA2012638	A/A	A/A	Genoscope *
Asiatic Mouflon	ERR157939	SAMEA2012639	A/A	A/D	Genoscope *
Asiatic Mouflon	ERR157942	SAMEA2012640	A/A	A/D	Genoscope *
Asiatic Mouflon	ERR157931	SAMEA2012641	A/A	D/D	Genoscope *
Asiatic Mouflon	ERR157932	SAMEA2012642	A/A	A/A	Genoscope *
Asiatic Mouflon	ERR157944	SAMEA1967031	A/A	A/A	Genoscope *
Asiatic Mouflon	ERR157935	SAMEA2012643	A/A	A/A	Genoscope *
Asiatic Mouflon	ERR332589	SAMEA2065600	A/A	A/A	Genoscope *
Asiatic Mouflon	ERR332575	SAMEA2065601	A/A	D/D	Genoscope *
Asiatic Mouflon	ERR332587	SAMEA2065602	A/A	A/A	Genoscope *
Asiatic Mouflon	ERR332582	SAMEA2065603	A/A	A/A	Genoscope *
Asiatic Mouflon	ERR332573	SAMEA1972234	A/A	A/D	Genoscope *
Asiatic Mouflon	ERR315509	SAMEA2065604	A/A	A/A	Genoscope *
Asiatic Mouflon	ERR466546	SAMEA2395410	A/A	A/A	Genoscope *
Asiatic Mouflon	ERR466544	SAMEA2395411	A/A	A/A	Genoscope *
Finnsheep	SRR11657543	SAMN14590314	A/A	A/A	Li, et al. [2]
Finnsheep	SRR11657544	SAMN14590313	A/D	A/D	Li, et al. [2]
Finnsheep	SRR11657545	SAMN14590312	A/A	A/A	Li, et al. [2]
Finnsheep	SRR11657546	SAMN14590311	A/A	A/A	Li, et al. [2]
Romanov	SRR12396891	SAMN15517583	A/A	A/D	Deng, et al. [3]
Romanov	SRR4291219	SAMN05216760	A/A	A/A	Heaton, et al. [4]
Romanov	SRR4291223	SAMN05216759	A/A	D/D	Heaton, et al. [4]
Romanov	SRR4291160	SAMN05216766	A/A	A/A	Heaton, et al. [4]
Swiss White Alpine	ERR3086436	SAMEA5239874	D/D	D/D	University of Bern**
Swiss White Alpine	ERR3086440	SAMEA5239878	D/D	D/D	University of Bern**
Swiss White Alpine	ERR3086476	SAMEA5239914	A/D	A/D	University of Bern**
Swiss White Alpine	ERR3086477	SAMEA5239915	A/D	A/D	University of Bern**
Rambouillet	SRR4291242	SAMN05216757	D/D	D/D	Heaton, et al. [4]
Rambouillet	SRR4291257	SAMN05216755	***	D/D	Heaton, et al. [4]
Rambouillet	SRR4291268	SAMN05216753	D/D	D/D	Heaton, et al. [4]
Rambouillet	SRR6305143	SAMEA104496890	A/D	A/D	Baylor College of Med.
Ancient DNA Seq	ERR3861593	SAMEA6516192	***	A/A	Yurtman, et al. [5]
Ancient DNA Seq	ERR3861592	SAMEA6516191	***	A/A	Yurtman, et al. [5]
Ovis ammon	SRR8560952	SAMN10915547	A/A	A/A	CAAS****
Ovis ammon	SRR8560953	SAMN10915548	A/A	A/A	CAAS****
Ovis ammon	SRR9222805	SAMN11979390	A/A	A/A	CAAS****
Ovis ammon	SRR9222806	SAMN11979389	A/A	A/A	CAAS****
Ovis ammon	SRR9222807	SAMN11979391	A/A	A/A	CAAS****
Ovis canadensis	SRR501858	SAMN01000748	A/A	A/A	Baylor College of Med.
Ovis canadensis	SRR501895	SAMN01000746	A/A	A/A	Baylor College of Med.

Ovis canadensis	SRR501898	SAMN01000747	A/A	A/A	Baylor College of Med.
Ovis dalli	SRR501847	SAMN01000785	A/A	A/A	Baylor College of Med.
Ovis dalli	SRR501897	SAMN01000764	A/A	A/A	Baylor College of Med.
Ovis vignei	ERR454945	SAMEA2358291	A/A	A/A	Genoscope *
Ovis vignei	ERR454946	SAMEA2358287	A/A	A/A	Genoscope *
Ovis vignei	ERR454947	SAMEA2358290	A/A	A/A	Genoscope *
Ovis vignei	ERR454948	SAMEA2358289	A/A	A/A	Genoscope *
Ovis vignei	ERR454950	SAMEA2358291	A/A	A/A	Genoscope *
Ovis nivicola	ERR4161992	SAMEA6833340	A/A	A/A	Upadhyay, et al. [6]
Ovis nivicola	ERR6667562	SAMEA8657697	A/A	A/A	Upadhyay, et al. [7]
Ovis nivicola	ERR6668200	SAMEA8657699	A/A	A/A	Upadhyay, et al. [7]
Ovis nivicola	ERR6668794	SAMEA8657700	A/A	A/A	Upadhyay, et al. [7]
Ovis nivicola	ERR6667561	SAMEA8657698	A/A	A/A	Upadhyay, et al. [7]
Ovis nivicola	ERR5858461	SAMEA8657696	A/A	A/A	Upadhyay, et al. [7]

* Sequenced as part of the NextGen project
** Institute of Genetics
*** No reads mapped
**** Institute of Animal Science of CAAS

Supplementary Table S5: SNP variants of rs413316737 observed in different breeds, also shown is the tail phenotype and the genotype frequency count in animals submitted by "the International Sheep Genome Consortium" and animals present in the house-own database. Breeds for which the investigated phenotype is not known were removed

Breed	Trait (Tail)	Sum			In-house SNP-Chip genotyping Data
		G/G	G/C	C/C	
Afshari	fat-tailed	1	1	0	0
Awassi	fat-tailed	1	0	2	0
Bangladeshi	short and thin	0	0	2	0
Castellana	long and thin	1	1	0	0
Cheviot	long and thin	2	0	0	0
Changthangi	short and thin	0	1	1	0
Churra	long and thin	2	0	0	0
Coopworth	long and thin	30	5	2	0
D'Man	long and thin	20	3	3	0
Finnsheep	short and thin	2	6	11	15
Garut	thin-tailed	1	1	0	0
Gulf Coast Native	long and thin	2	0	0	0
Karakas	fat-tailed	0	1	1	0
Merino Horned	long and thin	1	3	0	0
Merino Polled	long and thin	3	3	0	0
Merino	long and thin	2	1	0	0
Norduz	fat-tailed	1	1	0	0
Norwegian White Sheep	short and thin	2	0	0	0
Ojalada	long and thin	1	1	0	0
Ouled Djellal	long and thin	1	2	5	0
Romney	long and thin	40	3	2	0
Ronderib Afrikaner	fat-tailed	0	1	1	0
Salz	short and thin	0	1	1	0
Santa Ines	thin-tailed	0	1	1	0
Sardinian Ancestral Black	long and thin	19	5	0	0
Sumatran	short and thin	0	0	2	0
Swiss White Alpine	long and thin	25	2	0	24
Texel	short and thin	12	14	8	24
Wiltshire	long and thin	2	0	0	0
Alai	fat rumped	7	12	1	20
Aykol	fat rumped	1	7	11	19
Gissar	fat rumped	1	12	7	20
Kerry Hill	hock length	2	0	0	2
Alpines Steinschaf	hock length	5	10	1	16
Merinolandschaf	long and thin	225	161	72	458
Asiatic Mouflon	short and thin	12	3	2	17
Tien-Shan	long and thin	6	13	1	20
Berrichon	long and thin	19	0	0	19
Blanc du Massif Central	long and thin	19	1	0	20
Causses du Lot	long and thin	20	0	0	20

Mouton Charollais	long and thin	24	0	0	24
Charmoise	long and thin	23	0	0	23
Corse	long and thin	12	4	0	16
European Mouflon	short and thin	0	2	0	2
Île de France	long and thin	23	0	0	23
Limousine	long and thin	18	0	0	18
Lacaune meat	long and thin	33	1	0	34
Lacaune milk	long and thin	36	0	0	36
Mérinos d'Arles	long and thin	12	6	0	18
Mourerous	long and thin	15	1	0	16
Manech Tête Rouge	long and thin	22	3	0	25
Noire du Velay	long and thin	16	3	0	19
Ouessant	short and thin	4	6	8	18
Préalpes du Sud	long and thin	15	2	0	17
Rava	long and thin	20	0	0	20
Mérinos de Rambouillet	long and thin	0	2	25	27
Roussin de la Hague	long and thin	21	0	0	21
Romanov	short and thin	2	7	5	14
Rouge de l'Ouest	long and thin	17	0	0	17
Tarasconnaise	long and thin	14	1	0	15
Mouton Vendéen	long and thin	22	0	0	22
Dalapaels(Dala fur)	short and thin	0	1	20	21
Fjaellnaes	short and thin	10	0	0	10
Gotland	short and thin	11	7	1	19
Gute	short and thin	11	11	0	22
Kloevsjoe	short and thin	14	7	0	21
Swiss Mirror Sheep	long and thin	19	0	0	19
Valais Blacknose Sheep	long and thin	24	0	0	24
Valais Red Sheep	long and thin	24	0	89	113
Soay	short and thin	1	50	137	188
Argali	short and thin	0	0	5	5
Canadensis (Bighorn)	short and thin	0	0	3	3
Dalli	short and thin	0	0	2	2
Urial	short and thin	0	0	5	5
SnowSheep	short and thin	0	0	6	6
Cyprus Mouflon	short and thin	0	0	5	5
Jezersko-Solcava sheep	long and thin	33	3	0	36

The last column shows the number of animals from the in-house database from which the genotypes at the mentioned position were extracted. This database contains the samples genotyped in house and additionally, unfiltered genotype data recieved by the Vetsuisse Faculty, Institute of Genetics, University of Bern; online available genotype data from:

- Rochus et al, BMC Genomics, 2018 (https://doi.org/10.1186/s12864-018-4447-x)
- Rochus et al, BMC Genet, 2020 (DOI: 10.1186/s12863-020-0827-8)
- Stoffel et al, Nat Commun 2021 (10.1038/s41467-021-23222-9)

and genotypes extracted from online available Whole-Genome-Sequences with following run numbers and BioSample identifiers :

Ovis canadensis	SRR501858	SAMN01000748	Baylor College of Med.
Ovis canadensis	SRR501895	SAMN01000746	Baylor College of Med.
Ovis canadensis	SRR501898	SAMN01000747	Baylor College of Med.
Ovis dalli	SRR501847	SAMN01000785	Baylor College of Med.
Ovis dalli	SRR501897	SAMN01000764	Baylor College of Med.
Ovis vignei	ERR454945	SAMEA2358291	Genoscope *
Ovis vignei	ERR454946	SAMEA2358287	Genoscope *
Ovis vignei	ERR454947	SAMEA2358290	Genoscope *
Ovis vignei	ERR454948	SAMEA2358289	Genoscope *
Ovis vignei	ERR454950	SAMEA2358291	Genoscope *
Ovis nivicola	ERR4161992	SAMEA6833340	Upadhyay, et al. [6]
Ovis nivicola	ERR6667562	SAMEA8657697	Upadhyay, et al. [7]
Ovis nivicola	ERR6668200	SAMEA8657699	Upadhyay, et al. [7]
Ovis nivicola	ERR6668794	SAMEA8657700	Upadhyay, et al. [7]
Ovis nivicola	ERR6667561	SAMEA8657698	Upadhyay, et al. [7]
Ovis nivicola	ERR5858461	SAMEA8657696	Upadhyay, et al. [7]

* Sequenced as part of the NextGen project

Supplementary Table S6: Presents descriptive statistics about all measured and noted traits of all 362 phenontyped lambs

	Min-imum	1st quantile	median	mean	3rd quantile	Max-imum
**Age (days)**	3.00	25.00	28.00	28.99	35.00	53.00
**Body weight (kg)**	5.3	11.03	13.5	13.86	16.48	26.1
**Withers height (cm)**	34	46.8	49.5	49.42	52.3	57.7
**Tail length (cm)**	14.2	24.4	27.85	29.12	34.85	40.8

Supplementary Table S7: The first primer pair was used to genotype all 362 lambs and their fathers for the SNP located on OAR11:37,290,361 (Oar_v4.0), the second one was used to genotype the insertion (SV), localized within the promotor region of *HOXB13*. All other primer pairs, ordered for genotyping the SV did not fulfill our quality criteria

Primerpair	Sense Primer	AntiSensePrimer
To genotype SNP	TTTAAAACGCTTTGGATT	CACTCGGCAGGAGTAGTA
To genotype SV	TTTATGAGCTTCTCTCCGCCA	CACTCGGCAGGAGTAGTA
To genotype SV	TTTATGAGCTTCTCTCCGCCA	GACACTCGGCAGGAGTAGTAC
To genotype SV	GGAAGGAGGAGGGAGTGAGC	CTCCCAGCAAGCCTTCAATC
Used in Li, et al. [8] for SV	TTTATGAGCTTCTCTCCGCCA	AAGTGGTATAATTGCCGGGCT
To genotype SV	TTTATGAGCTTCTCTCCGCCA	TGGTATAATTGCCGGGCTCC

Supplementary Methods

Determining Candidate SNPs and small indels based on all available sequences

We built a gvcf database[9] from these BAM files:

(1) 2 pooled samples, representing short-tailed group and long-tailed group of our 48 Merinolandschaf lambs,
(2) 37 wild sheep samples,
(3) 16 NCBI samples, representing 8 samples of short-tailed breeds and long-tailed breeds each.

Next, we performed SNP and small indels calling using standard GATK practices. However, in this step, we did not perform any filtering of variants because we wanted to keep all possible variants in the candidate region. Finally, we identified candidate variants following these steps:

(1) The ancestral allele was determined based on 37 wild sheep samples; here, the major allele was set as an ancestral allele.
(2) If the ancestral allele was determined successfully in step (1), it was checked whether the number of ancestral sheep containing the derived allele was less than or equals to 5. This soft threshold of the derived allele was set as such to take into account the possibility of introgression between domestic sheep and wild sheep.
(3) It was determined if the read counts supporting reference alleles and the read counts supporting alternate alleles is significantly different (Fisher exact test, P-value<0.05 after multiple test correction) between the pooled sample of short-tailed group and long-tailed group.
(4) If the P-value as estimated in step (3) was significant, it was determined if the allele counts of reference and alternate allele is significantly different (Fisher exact test, P-value<0.05 after multiple test correction) between the NCBI samples of short-tailed group and long-tailed group.
(5) Finally, we checked if the number of reads supporting derived alleles is significantly higher than the number of ancestral alleles in the pooled sample of the long-tailed group.

This resulted in 19 variants seen in Supplementary Table S6. We then examined these 19 variants in our 48 sequenced lambs separately. Because their BAM files had low coverage, we

selected only those variants that were 90 % present in individuals that had mapped reads at that particular position in one group and no more than 10 % in the other group. Additionally all 19 variants were determined with the Ensembl Variant Effect Predictor[1] after remapping to Oar_3.1 using NCBI remap (results of the Determination are presented in Supplementary Table S4). No variant met the above-mentioned criterion, but since the missense variant with ID rs413316737 was very close to the threshold and it reside within the first exon of our candidate gene, we decided to investigate it further.

Supplementary References

1 McLaren, W. *et al.* The Ensembl Variant Effect Predictor. *Genome biology* **17**, 122 (2016). https://doi.org:10.1186/s13059-016-0974-4

2 Li, X. *et al.* Whole-genome resequencing of wild and domestic sheep identifies genes associated with morphological and agronomic traits. *Nat Commun* **11**, 2815 (2020). https://doi.org:10.1038/s41467-020-16485-1

3 Deng, J. *et al.* Paternal Origins and Migratory Episodes of Domestic Sheep. *Curr Biol* **30**, 4085-4095 e4086 (2020). https://doi.org:10.1016/j.cub.2020.07.077

4 Heaton, M. P. *et al.* Using sheep genomes from diverse U.S. breeds to identify missense variants in genes affecting fecundity. *F1000Res* **6**, 1303 (2017). https://doi.org:10.12688/f1000research.12216.1

5 Yurtman, E. *et al.* Archaeogenetic analysis of Neolithic sheep from Anatolia suggests a complex demographic history since domestication. *Commun Biol* **4**, 1279 (2021). https://doi.org:10.1038/s42003-021-02794-8

6 Upadhyay, M. *et al.* The First Draft Genome Assembly of Snow Sheep (Ovis nivicola). *Genome Biol Evol* **12**, 1330-1336 (2020). https://doi.org:10.1093/gbe/evaa124

7 Upadhyay, M. *et al.* Whole genome sequencing reveals a complex introgression history and the basis of adaptation to subarctic climate in wild sheep. *Mol Ecol* **30**, 6701-6717 (2021). https://doi.org:10.1111/mec.16184

8 Li, R. *et al.* The first sheep graph-based pan-genome reveals the spectrum of structural variations and their effects on tail phenotypes. (2022). https://doi.org:10.1101/2021.12.22.472709

9 Auwera, G. d. & O'Connor, B. *Genomics in the Cloud: Using Docker, GATK, and WDL in Terra*. 1st Edition edn, (O'Reilly Media, 2020).
