# Supplementary material for: Fine-mapping and identification of candidate causal genes for tail length in the Merinolandschaf breed
Source: Commun Biol. 2022 Sep 6;5:918. doi: 10.1038/s42003-022-03854-3 (PMC9448734; doi:10.1038/s42003-022-03854-3)
Supplement: Supplementary file 2 — Reporting Summary [file 42003_2022_3854_MOESM2_ESM.pdf]

nature research

Corresponding author(s): Ivica Medugorac

Last updated by author(s): Jul 14, 2022

Reporting Summary

Nature Research wishes to improve the reproducibility of the work that we publish. This form provides structure for consistency and transparency in reporting. For further information on Nature Research policies, see our Editorial Policies and the Editorial Policy Checklist.

Statistics

For all statistical analyses, confirm that the following items are present in the figure legend, table legend, main text, or Methods section.

n/a	Confirmed	
☐	☒	The exact sample size (*n*) for each experimental group/condition, given as a discrete number and unit of measurement
☐	☒	A statement on whether measurements were taken from distinct samples or whether the same sample was measured repeatedly
☐	☒	The statistical test(s) used AND whether they are one- or two-sided<br>*Only common tests should be described solely by name; describe more complex techniques in the Methods section.*
☐	☒	A description of all covariates tested
☐	☒	A description of any assumptions or corrections, such as tests of normality and adjustment for multiple comparisons
☐	☒	A full description of the statistical parameters including central tendency (e.g. means) or other basic estimates (e.g. regression coefficient) AND variation (e.g. standard deviation) or associated estimates of uncertainty (e.g. confidence intervals)
☐	☒	For null hypothesis testing, the test statistic (e.g. *F*, *t*, *r*) with confidence intervals, effect sizes, degrees of freedom and *P* value noted<br>*Give P values as exact values whenever suitable.*
☒	☐	For Bayesian analysis, information on the choice of priors and Markov chain Monte Carlo settings
☒	☐	For hierarchical and complex designs, identification of the appropriate level for tests and full reporting of outcomes
☐	☒	Estimates of effect sizes (e.g. Cohen's *d*, Pearson's *r*), indicating how they were calculated

Our web collection on statistics for biologists contains articles on many of the points above.

Software and code

Policy information about availability of computer code

Data collection	The NCBI Sequence Read Archive was used to download whole genome sequences of muliple domesticated and wild sheep
Data analysis	GCTA v1.93.2<br>ASReml 4.1<br>Beagle 5.0<br>Enrichr<br>R 4.1.2<br>Sickle 1.33<br>FastQC v0.11.8<br>BWA-MEM 0.7.17-r1198-dirty<br>Samtools 1.12<br>Picard 2.21.1-SNAPSHOT<br>GATK 3.8<br>Lumpy v0.2.13<br>Delly v0.8.5<br>Jbrowse 2<br>Primer3Plus<br>SnapGene Viewer 6.0.2

For manuscripts utilizing custom algorithms or software that are central to the research but not yet described in published literature, software must be made available to editors and reviewers. We strongly encourage code deposition in a community repository (e.g. GitHub). See the Nature Research guidelines for submitting code & software for further information.

Data

Policy information about availability of data

All manuscripts must include a data availability statement. This statement should provide the following information, where applicable:

- Accession codes, unique identifiers, or web links for publicly available datasets
- A list of figures that have associated raw data
- A description of any restrictions on data availability

Genotype and phenotype data as well as Sanger sequences that support the findings of this study have been deposited in Figshare (https://doi.org/10.6084/m9.figshare.19375712, https://doi.org/10.6084/m9.figshare.19368962, https://doi.org/10.6084/m9.figshare.19368905, https://doi.org/10.6084/m9.figshare.19368734). Capture sequencing data have been deposited in European Nucleotide Archive (ENA) with the study accession PRJEB51698. All other data are available from the corresponding author on reasonable request.

Field-specific reporting

Please select the one below that is the best fit for your research. If you are not sure, read the appropriate sections before making your selection.

☐ Life sciences ☐ Behavioural & social sciences ☒ Ecological, evolutionary & environmental sciences

For a reference copy of the document with all sections, see nature.com/documents/nr-reporting-summary-flat.pdf

Ecological, evolutionary & environmental sciences study design

All studies must disclose on these points even when the disclosure is negative.

Study description	This study aimed to analyze the natural tail length variation in the Merinolandschaf and to identify causal alleles for the short tail phenotype segregating within long-tailed breeds. We used SNP-based association analysis and haplotype-based mapping in 362 genotyped (Illumina OvineSNP50) and phenotyped Merinolandschaf lambs
Research sample	The researched animals were lambs of the Merinolandschaf breed (Ovis aries).
Sampling strategy	No sample size calculation was performed. We performed selective phenotyping, sampling and genotyping on 96 beaches (format of microtitre plates and Illumina OvineSNP50 chip). The number of 384 samples (including sires) achieved genome-wide significant results.
Data collection	We performed selective phenotyping, i.e. phenotyping and sampling of phenotypic extremes from a large, visually inspected population. The phenotypic data were collected by the veterinarians Kim Eck and Elisabeth Hannemann with the support of a technician. The data were entered into forms specially developed for this purpose.
Timing and spatial scale	Data were collected in Germany in November and December 2017; February, March and November 2018; March and December 2019
Data exclusions	No data were excluded
Reproducibility	The Reproducibility of phenotypes was investigated in the pilot study performed and published by Eck et al (DOI10.1016/j.smallrumres.2019.04.007). The reproducibility of SNP genotyping is ensured by the reproducibility of the Illumina chip technology.
Randomization	As described by DARVASI and SOLLER (1992) for selective genotyping, mainly lambs with extremely short or extremely long tails were considered
Blinding	Because our study aimed at identifying the genetic basis of the long-tailed phenotype in Merinolandschaf breed, blinding is not relevant here.

Did the study involve field work? ☒ Yes ☐ No

Field work, collection and transport

Field conditions	Sampling and phenotyping took place in stalls and were done by veterinarians and a technical assistant; weather conditions were not relevant for the results
Location	Sheep farm in Lover Bavaria and in the teaching and research farm “Oberer Hardthof” at the Justus Liebig University of Giessen
Access & import/export	All blood samples were taken according to best veterinary practice and under a permit from the Government of Upper Bavaria (permit number: 55.2-1-54-2532.0-47-2016), or the Regional Council of Gießen, Hassia (KTV number: 19 c 20 15 h 02 Gi 19/1 KTV 22/2020).
Disturbance	NA

Reporting for specific materials, systems and methods

We require information from authors about some types of materials, experimental systems and methods used in many studies. Here, indicate whether each material, system or method listed is relevant to your study. If you are not sure if a list item applies to your research, read the appropriate section before selecting a response.

Materials & experimental systems

n/a	Involved in the study
☒	☐ Antibodies
☒	☐ Eukaryotic cell lines
☒	☐ Palaeontology and archaeology
☐	☒ Animals and other organisms
☒	☐ Human research participants
☒	☐ Clinical data
☒	☐ Dual use research of concern

Methods

n/a	Involved in the study
☒	☐ ChIP-seq
☒	☐ Flow cytometry
☒	☐ MRI-based neuroimaging

Animals and other organisms

Policy information about studies involving animals; ARRIVE guidelines recommended for reporting animal research

Laboratory animals	The study did not involve laboratory animals.
Wild animals	Only open, already available wildlife source data were included in this study.
Field-collected samples	The lambs studied were kept on farms and were approximately 5 weeks old.
Ethics oversight	The collection of blood samples for this study was approved by the ethics committee of the Veterinary Faculty of LMU Munich. All blood samples were taken according to best veterinary practice and under a permit from the Government of Upper Bavaria (permit number: 55.2-1-54-2532.0-47-2016), or the Regional Council of Gießen, Hassia (KTV number: 19 c 20 15 h 02 Gi 19/1 KTV 22/2020).

Note that full information on the approval of the study protocol must also be provided in the manuscript.
